# Characterisation of the novel spontaneously immortalized and invasively growing human skin keratinocyte line HaSKpw

**DOI:** 10.1038/s41598-020-71315-0

**Published:** 2020-09-16

**Authors:** Elizabeth Pavez Lorie, Nicola Stricker, Beata Plitta-Michalak, I.-Peng Chen, Beate Volkmer, Rüdiger Greinert, Anna Jauch, Petra Boukamp, Alexander Rapp

**Affiliations:** 1 Leibniz Research Institute for Environmental Medicine, Auf’m Hennekamp 50, 40225 Düsseldorf, Germany; 2 Cell Biology and Epigenetics, Department of Biology, Technical University of Darmstadt, Schnittspahnstr. 10, 64287 Darmstadt, Germany; 3 Centre of Dermatology, Elbe Clinics, Am Krankenhaus 1, Buxtehude, 21614 Germany; 4 Institute of Human Genetics, University Heidelberg, 69120 Heidelberg, Germany

**Keywords:** Skin cancer, Squamous cell carcinoma, Cell biology

## Abstract

We here present the spontaneously immortalised cell line, HaSKpw, as a novel model for the multistep process of skin carcinogenesis. HaSKpw cells were established from the epidermis of normal human adult skin that, without crisis, are now growing unrestricted and feeder-independent. At passage 22, clonal populations were established and clone7 (HaSKpwC7) was further compared to the also spontaneously immortalized HaCaT cells. As important differences, the HaSKpw cells express wild-type p53, remain pseudodiploid, and show a unique chromosomal profile with numerous complex aberrations involving chromosome 20. In addition, HaSKpw cells overexpress a pattern of genes and miRNAs such as KRT34, LOX, S100A9, miR21, and miR155; all pointing to a tumorigenic status. In concordance, HaSKpw cells exhibit reduced desmosomal contacts that provide them with increased motility and a highly migratory/invasive phenotype as demonstrated in scratch- and Boyden chamber assays. In 3D organotypic cultures, both HaCaT and HaSKpw cells form disorganized epithelia but only the HaSKpw cells show tumorcell-like invasive growth. Together, HaSKpwC7 and HaCaT cells represent two spontaneous (non-genetically engineered) “premalignant” keratinocyte lines from adult human skin that display different stages of the multistep process of skin carcinogenesis and thus represent unique models for analysing skin cancer development and progression.

## Introduction

Cell lines are important tools for effectively reproducing and investigating biological processes or diseases and have been a crucial tool in cancer research. One key initial step in the establishment of cell lines is the process of immortalisation^1^, providing the cells with indefinite growth capacity. Accordingly, many keratinocyte cell lines used in dermatological research are based on immortalization by either SV40^2–6^ or HPV^7^ and later by introducing the hTERT gene (human telomerase reverse transcriptase), when it was clear that telomerase activation was required for continuous growth^8^.

Alternatively, spontaneous immortalisation represents an unbiased endogenously driven mechanism. Although spontaneous immortalization of human cells is rare, some keratinocyte lines have been described already, mainly derived from neonatal foreskin (for review see Allen-Hoffmann et al.^9^). Genetic analysis of the best characterized cell line, the NIKS cells, demonstrated an extra copy of i(8q) as the only and consistent karyotypic abnormality^9^. As chromosome 8q harbours the proto-oncogenic transcription factor c-myc, known for its essential role in cellular growth control, cell transformation, and tumorigenesis^10^, the presence of an extra i(8q) was thought to be causally related with the immortalization process. Alternatively, the spontaneously immortalized HaCaT cell line was derived from adult trunk skin^11^ and thus may represent a different evolutionary pathway. Its genetic profile proved to be quite different, though still reflecting chromosomal aberrations characteristic for cutaneous squamous-cell carcinomas (cSCC). Furthermore, the HaCaT cells show UV-indicative p53 mutations characteristic not only for cSCC, but also frequently detected in normal human skin^12^. Together, the HaCaT cells with their specific genetic profile, carry changes occurring in the epidermis in vivo and thus represent a relevant model for an early stage of skin carcinogenesis.

We now describe a second keratinocyte cell line established spontaneously from a keratinocyte culture of adult trunk skin. Interestingly, and different from most other cell lines, in particular the HaCaT cells, the HaSKpw cells, which were established from “normal” skin keratinocytes without any sign of growth retardation or crisis present a unique spectrum of genetic and growth properties. It is tempting to propose that this may reflect the broad spectrum of events occurring in human skin and contributing to the frequent development of actinic keratosis and finally cSCC. As also these cells were not genetically engineered, they may well add to an unbiased view of the broad spectrum of early genetic events occurring in naïve human skin and contributing to the genetic heterogeneity of AKs and cSCCs.

## Results

### Development of the HaSKpw cell line

Primary keratinocytes were isolated from full thickness adult human skin and plated at high cell density on a feeder layer, as described in Materials and Methods. The first passage reached 90% confluence after 8 days. After passage 8 the cells were continuously frozen and stored. Cells were successfully passaged using the regular EDTA/trypsin (0.05%/0.4%) subculture protocol. These cells continued to grow without showing sign of growth retardation or even entering a cell crisis, suggesting an early gain of unrestricted growth potential. During subsequent passaging the keratinocytes became independent of feeder support and were henceforward cultured in FAD medium with 10% FCS at a split ratio of 1:10. As the HaSKpw cells represented a genetically unstable mass population, likely consisting of a number of subpopulations, the cells were cloned at passage 22, with clone number 7 (HaSkpwC7) being further propagated and used for forthcoming experiments.

To determine the identity/singularity of this newly generated skin keratinocyte line, Short Tandem Repeat (STR) profiles were generated from passage 1, 7 and 33, the latter representing HaSKpwC7 cells. The resulting STR profile proved to be unique and of human origin with no matches in any of the following databases: ATCC (USA), HPACC (UK), JCRB (Japan), RIKEN (Japan), KCLB (Korea) and DSMZ (Germany). The profile is listed in Table 1 together with the STR profile of the spontaneously immortalised human HaCaT keratinocyte line. The new cell line was verified to be negative for Hepatitis B and C, HIV 1 and 2 as well as mycoplasma. The cell line was named HaSKpw, indicating its origin; human adult spontaneously immortalized keratinocytes, p53 wildtype (see below).

**Table 1:** STR profile (with the different human specific genetic site) of HaSKpwC7 and HaCaT cell lines.

	D13S317	D5S818	D7S820	D16S539	vWA	THO1	TPOX	CSF1PO	Amelogenin
HaCaT	10,12	12,12	9,11	9,12	16,17	9.3, 9.3	11,12	9,11	X,X
HaSKpw^a^	11,11	11,11	10,12	12,13	14,17	6,7	8,11	10,11	X,X

^a^The STR profiles are identical for all three tested passages of HaSKpw cells, including the HaSKpwC7 cells and therefore only one result is listed.

### HaSKpw cells show an elevated telomerase activity and stable telomere length

**Figure 1:**
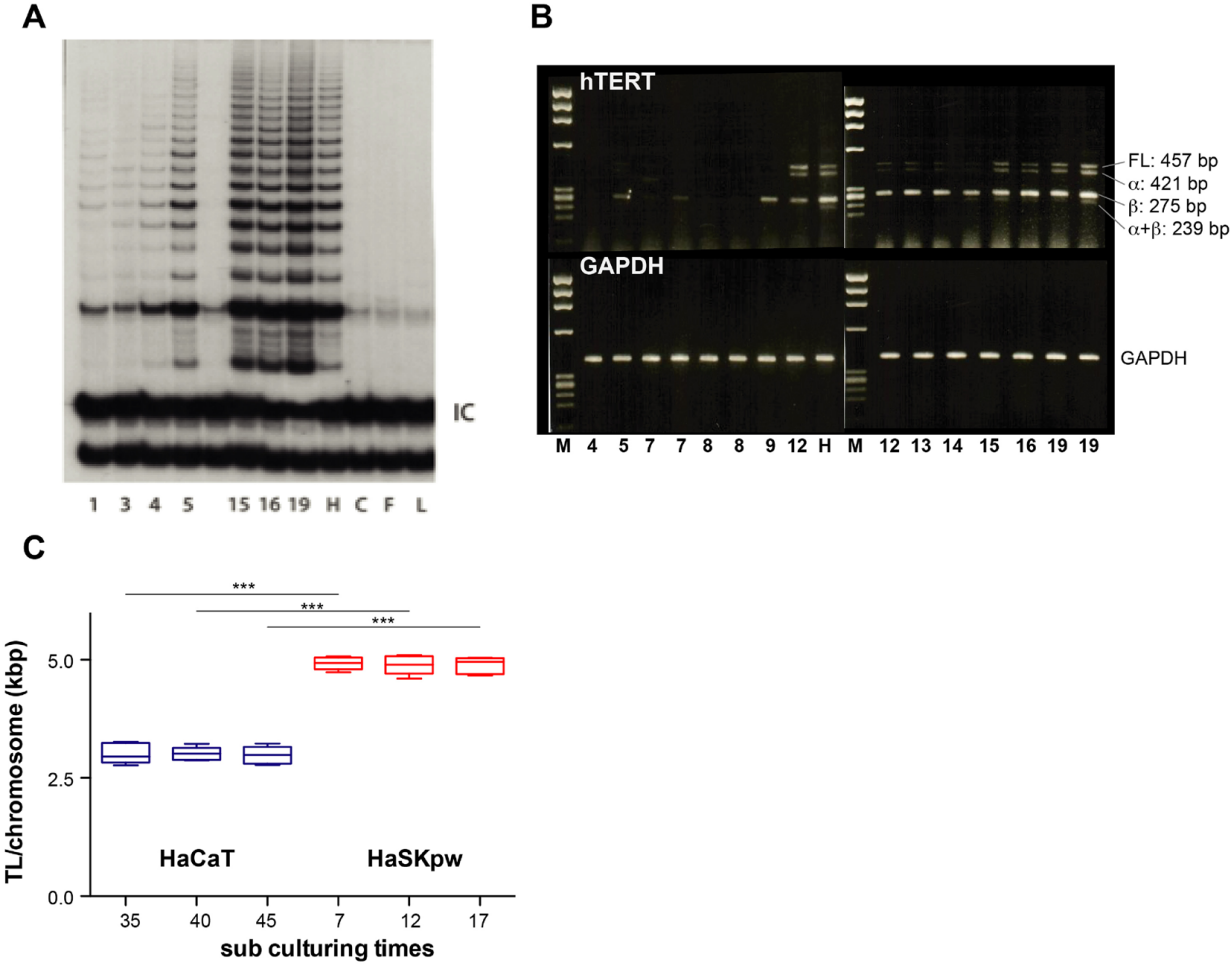
HaSKpw keratinocytes activate telomerase during immortalisation. (**A**) TRAP-Assay from different passage of the HaSKpw cells during the immortalisation process. The gradual increase in telomerase activity with passage number (1, 3, 4, 5, 15, 16, and 19) can be seen. HaCaT cells in a dilution of 1:10, were added as positive control and RNAse-treated HaCaT lysate (C), NHDF (F), and Lysis buffer (L) were added as negative controls. *IC* internal control. (**B**) Telomerase activity is dependent on the presence of the full-length splice variant of hTERT. Analysis of the expressed splice variants of hTERT in the HaSKpw keratinocytes during immortalisation. Splice variants were identified using cDNA generated from the cell line at the indicated passages. Primers amplify either full length or the α or β isoform of hTERT. Fragments were then identified on a 2% agarose gel. Marker (M), numbers indicate the passages of the HaSKpw cells during immortalisation. From passage 9 onwards the full-length (457 bp) splice variant becomes detectable. GAPDH was used as loading control. (**C**) Telomere length was measured for the HaSKpwC7 cells at passages 7, 12 and 17 and compared to HaCaT cells of passage 35, 40 and 45. Telomere length was measured in three biological replicates with three technical replicates for each every measurement point. Lines denote the median, boxes the 25th to 75th percentile, while whiskers are the 5th to 95th percentiles. Telomere length is stable during subculture. Significance was calculated by Anova and post hoc Tukey’s test; ***p < 0.001.

Telomerase activity and with that halted proliferation-dependent telomere loss is an essential survival mechanism, i.e. is essential for immortality^13^. The HaCaT cells that developed through survival of a single clone, expressed high level telomerase activity when first analysed at passage two^14^. Thus, up-regulation of telomerase activity was likely the initial event to allow cell survival of this monoclonal population. The HaSKpw cell line developed during subsequent propagation of the bulk population without any obvious crisis. This may therefore suggest that immortalization was not a rare event of a single cell but rather due to an intrinsic predisposition of the entire cell population. We previously showed that normal human skin keratinocytes (NHEK) are telomerase-positive in vivo, but that telomerase is down-regulated during in vitro passaging^15^. By performing the TRAP assays (telomeric repeat amplification protocol), we now show the status of telomerase activity in different passages of the HaSKpw cells (Fig. 1A). In accordance with our previous results on NHEK, also the HaSKpw cells showed only little telomerase activity during the first 4 passages. However, at passage 5, telomerase activity increased, reaching its maximum around passage 10. Compared to HaCaT cells, however, the level of telomerase activity remained reduced (about 10%) also in later passages of the HaSKpw as well as in HaSKpwC7 cells (Fig. 1A).

One mechanism of hTERT regulation is by alternative splicing^16^. Besides the only functional full-length transcript, the hTERT gene exhibits a number of splice variants with the α-splice form displaying a dominant-negative effect on telomerase activity and the β-splice form competing with full length hTERT for binding to hTR and that way suppressing telomerase activity. While normal cells predominantly express suppressive hTERT variants, a splicing switch to the full length active transcript was described as being characteristic for cancer^17^. When investigating different passages of the HaSKpw cells by RT-PCR, the early passage HaSKpw cells, if at all, expressed the ß splice variant. From passage 9 onwards, the splicing profile with full length (fl), α-, α/ß- and ß-splice form was seen with a prominent fl hTERT stabilizing around passage 12. (Fig. 1B). Together, this shows that the switch to stable “high” level telomerase activity occurred during passaging of the mass population between passages 9 and 12—which was verified repeatedly—and it is tempting to speculate that this was related to or even causal for the attainment of unrestricted growth, i.e. immortalization.

In the HaCaT cells, telomerase maintains a constant telomere length of ~ 3 kb also throughout long-term passaging. To determine whether the lower telomerase activity of the HaSKpw cells would also suffice for stabilization, telomere length was investigated for HaSKpwC7 and HaCaT cells by qPCR in passage intervals of 5. Quantification revealed a mean telomere length of 4.3 ± 0.12 kbp for HaSKpwC7 and 3.0 ± 0.20 kbp for HaCaT cells, respectively. This demonstrated that the HaSKpwC7 and HaCaT cells differ slightly in telomere length. Importantly, the individual telomere lengths were maintained unaltered upon continuous propagation, thereby, confirming sufficient functional telomerase also for HaSKpwC7 cells.

### HaSKpw cells are hypodiploid with several complex translocations

**Figure 2:**
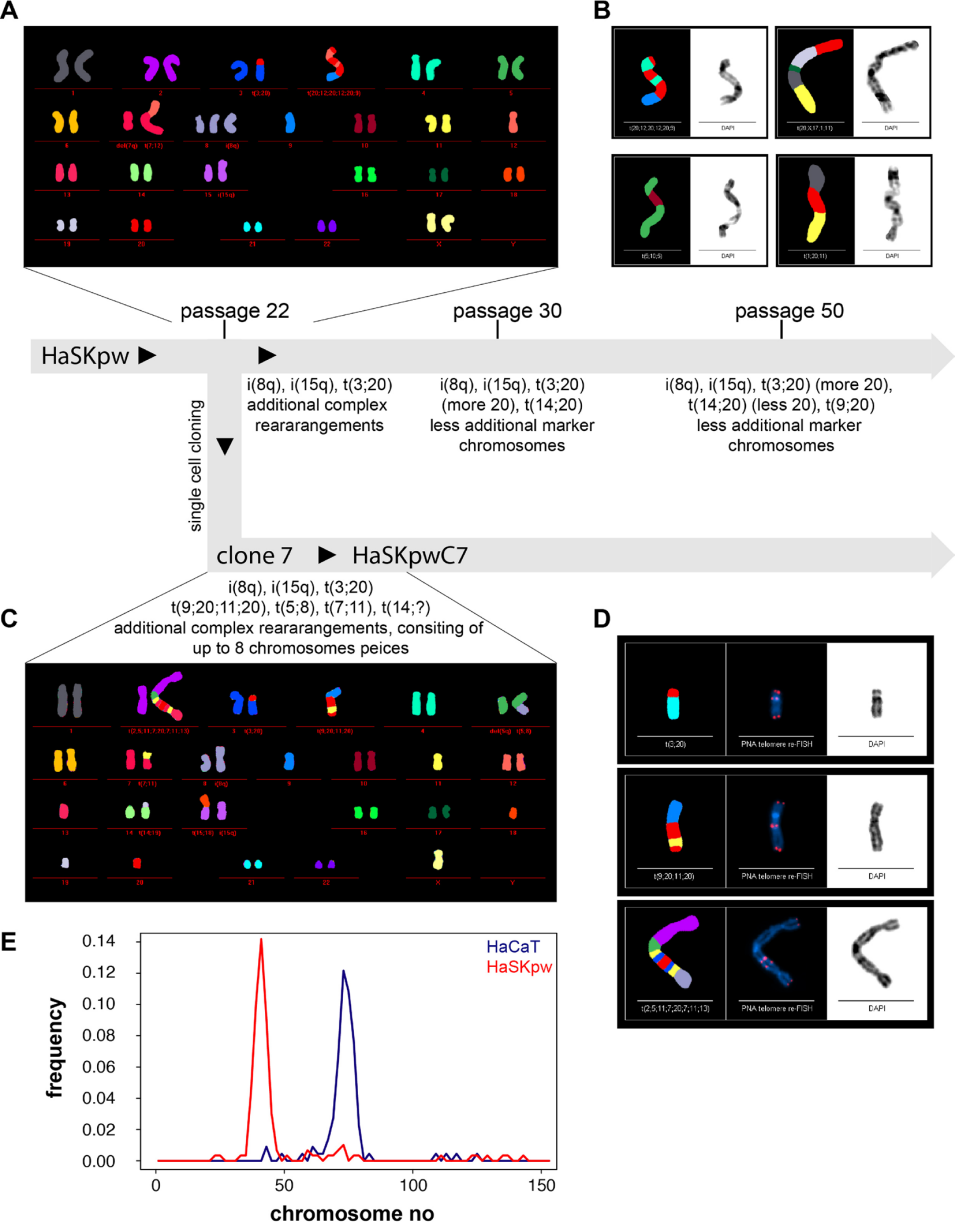
Karyotype evolution during the generation of the HaSKpw cell line. (**A**) The genetic development is schematically represented and M-FISH analysis were performed with HaSKpw cells at different passage levels. Highlighted are: (**A**) a representative karyotype of the HaSKpw cells at passage 22, the time of cell cloning as well as (**B**) examples of translocation chromosomes of the HaSKpw cells, carrying extra pieces of chromosome 20. Highlighted are also: (**C**) a representative karyotype of HaSKpwC7 cells and (**D**) examples of translocation chromosomes of the HaSKpwC7 cells, re-hybridized with a PNA probe for staining telomeres. Note the intra-chromosomal telomeres, likely deriving from chromosome 20. (**E**) Frequency analysis of metaphase spreads from HaSKpwC7 cells (passage 8–13) and HaCaT (passage 36–41) cells, indicating with a median chromosome number of 42 and 74, respectively.

Telomerase activity is supposed to be important for genomic stability. Accordingly, the HaCaT cells with their high telomerase activity showed a rather stable genotype/chromosomal constellation with distinct marker chromosomes characterizing the cell line also during long-term culture^11,18,19^. Noteworthy, while the HaCaT cells rapidly turned hypotetraploid, cytogenetic analyses of the HaSKpw cells demonstrated a rather stable pseudo-diploid karyotype (Fig. 2A,C). Polyploidization occurred only later in a subpopulation that never reached dominance. M-FISH analysis of the HaSKpw cells at passage 22 identified several dominant aberrations. Most metaphases contained 3 marker chromosomes t(3;20), i(8q), and i(15q). In addition, each metaphase contained up to 12 additional chromosomal aberrations with many complex translocations containing 2 to 6 pieces and frequently involving chromosome 20 (Fig. 2A,B). At later passages (≥ passage 30) a new marker chromosome occurred (t(14;20)) while the frequency of additional complex rearranged chromosomes was reduced.

Hoping for a genetically more stable subpopulation, HaSKpw cells were cloned at passage 22 and the HaSKpwC7 cells were further investigated. Besides the original marker chromosomes, these cells exhibited new markers (t(9;20;11;20), t(5;8), t(7;11), and t(14;?) (Fig. 2C) as well as many often highly complex translocation-chromosomes (with up to 8 chromosomal pieces) (Fig. 2D). Additional telomere staining demonstrated the presence of frequent intra-chromosomal telomeres with a high association of chromosome 20, suggesting that most intra-chromosomal telomeres in these complex chromosomal rearrangements were derived from the highly unstable chromosome 20 (Fig. 2D). Note, that also the HaSKpwC7 cells were found to be hypodiploid with a median chromosome number of 42 (Fig. 2E). Together, this demonstrates that despite distinct genomic instability, the HaSKpwC7 cells remain pseudodiploid, which is contrary to the HaCaT cells, and argues for differential genetic regulatory cues characterizing these two cell lines.

### HaSKpw cells have one functional wild type p53 allele

**Figure 3:**
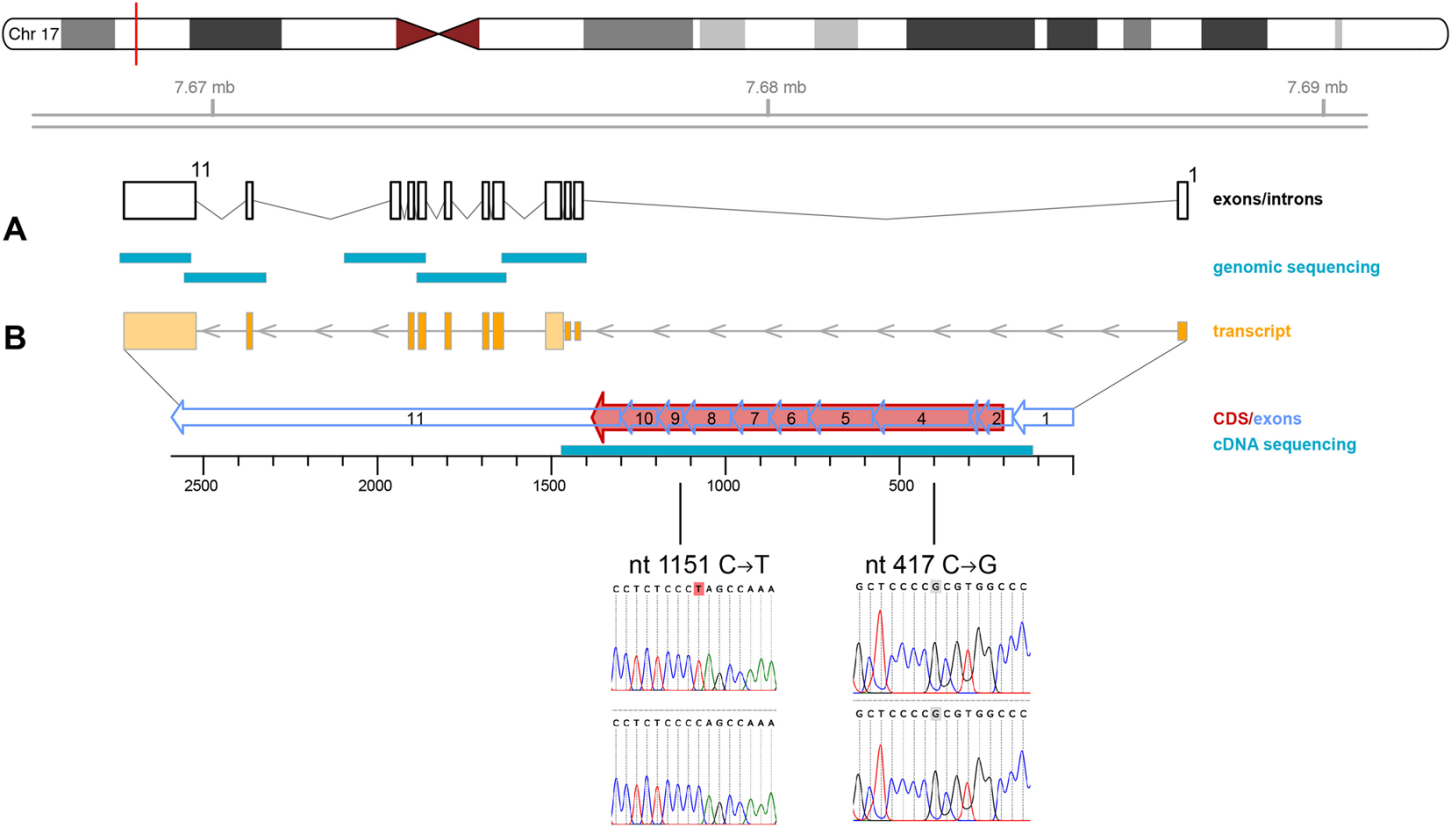
Analysis of the p53 locus in the HaSKpw cells. The p53 gene locus, located on chromosome 17 was sequenced by 5 overlapping amplicons (**A**) spanning exons 2–11 and including most of the intron sequences. (**B**) Sequencing of the p53 mRNA revealed a homozygous C–G polymorphism at nt position 417 and a heterozygous polymorphism at nt position 1,151 from C to T that results in a truncated version of p53. The analysis was performed with HaSKpwC7 (passage 10) and HaCaT (passage 39) cells, respectively.

One characteristic for skin cancer cells are UV-dependent mutations of the p53 gene^12,20^. In line with these observations, HaCaT cells exhibit UV-indicative mutations in both alleles of the p53 gene and these mutations were present already in the very early passages suggesting for an in vivo acquired mutation spectrum^21^. To determine the p53 status of the HaSKpw cells, the coding part of the genomic locus of p53 of the HaSKpwC7 cells was amplified and five amplicons were sequenced by “Sanger sequencing” (Fig. 3). Additionally, the mRNA sequence encoding for p53 of both HaCaT and HaSKpwC7 cells were cloned and sequenced. Thereby, we could confirm the two UV-indicative p53 mutations for the HaCaT cells. For the HaSKpwC7 cells, sequence analysis of the p53 mRNA revealed no mutations. However, besides one allele with wild-type p53 sequence, the HaSKpwC7 cells exhibited a heterozygous polymorphism at cDNA position 1,151 (Q328→*; dbSNP:rs764735889). This stop codon polymorphism results in a truncated and inoperable protein of approximately 34.8 kDa (Fig. S1). In addition, a homozygous polymorphism (417C→G; R72→P; dbSNP:rs1042522), present in approximately 35% of the human population, was found in HaSKpwC7 as well as in HaCaT cells (Fig. 3 and Supplementary Fig. S1). This polymorphism is located in the proline rich domain of p53 and is believed to correlate with reduced apoptosis and to be associated with increased cancer risks^22^.

These findings show that while HaCaT cells represent keratinocytes with mutant and therefore dysfunctional p53, the HaSKpwC7 cells express wt p53 and represent keratinocytes with functional p53 protein.

### HaSKpwC7 and HaCaT cells are similar in their proliferative properties, but differ in their population doubling times

**Figure 4:**
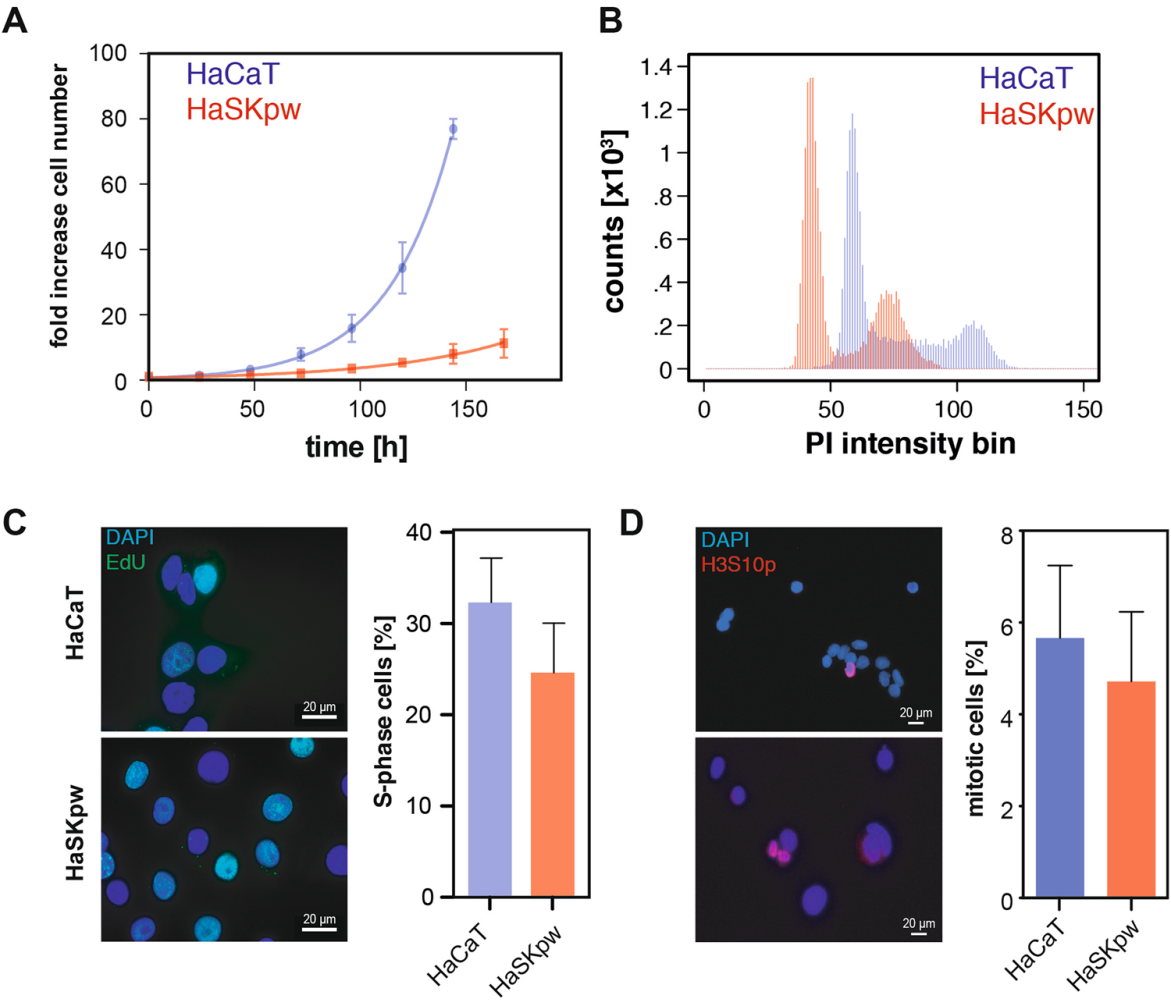
Growth characteristics of HaCaT and HaSKpwC7 cells. (**A**) Growth curve of HaCaT and HaSKpwC7 cells. Cells were counted and normalized. Points represent means of triplicates of three independent experiments and the error bars are standard deviation. (**B**) Cell cycle distribution for both cell lines. While the HaSKpwC7 cells exhibit a near diploid genome size, HaCaT cells show a 1.4-fold increase in genome size, with no significant differences in the distribution of the cell cycle phases. (**C**) Images of S-phase positive cells identified by EdU pulse labelling (green), for HaCaT cells (top) and HaSKpwC7 cells (down). Quantification of S-phase positive cells in both cell lines, compiled for each cell line from three independent biological replicates (mean values and standard deviation). (**D**) Image of HaCaT cells (top) and HaSKpwC7 cells (down) stained with anti-H3S10p (red) to identify mitotic cells. Quantification of the mitotic fractions is based on cell size and mean H3S10p intensity, compiled from two biological replicates (mean and standard deviation). All assays were performed with HaSKpwC7 cells of passage 9–14 and HaCaT cells of passage 37–43.

As a first step to understand the biological behaviour of the HaSKpwC7 cells, we studied their growth characteristics in comparison to the HaCaT cells. Our results demonstrate that both the HaSKpw and HaCaT show a lag phase of approximately 48 h, while during the exponential growth phase the HaCaT cell line shows faster cell growth with a population doubling time of 28.0 h compared to a population doubling time of 43.4 h calculated for the HaSKpwC7 cells (Fig. 4A). To understand this difference in more detail we analysed the cell cycle distribution of exponentially growing cultures by flow cytometry. Measuring the DNA content by quantitative DNA staining using propidium iodide (Fig. 4B), we could not detect significant differences in the cell cycle profiles of the HaCaT and HaSKpwC7 cells (Fig. 4C). While the HaCaT cells showed a more pronounced S-phase in the histogram, the HaSKpwC7 cells showed a more pronounced G2 peak. This analysis also confirmed the larger genome size of the HaCaT cells as compared to the HaSKpwC7 cells. Based on the intensity of the G1 peak, the HaCaT cells have a genome of approximately 1.45 times the size of HaSKpwC7 cells (Fig. 4B) (which reflects well the hypotetraploid versus pseudo-diploid state of the HaCaT and HaSKpwC7 cells, respectively, (see also Fig. 2).

To get further insight into the cell cycle distribution we performed pulse labelling of both cell lines using EdU (5-ethynyl-2′-deoxyuridine) pulses for 30 min to identify cells in S-Phase. Subsequently the cells were stained using the Click-IT chemistry and analysed using high content imaging. Figure 4C shows the HaCaT and HaSKpwC7 cells, where green-labelled cells represent cells in S-phase. Subsequently cells were gated for high EdU signal intensity and DAPI levels below the G2 peak to exclude cells that left S-phase and went into G2. Quantitative comparison of the two cell lines revealed non-significant difference with 32% S-phase cells for HaCaT and 26% for HaSKpwC7 cells respectively. We then focused on a detailed analysis of mitotic cells where we used an antibody specific for H3S10 (histone H3 Serine 10) phosphorylation. Exponentially growing cells were stained with the anti-H3S10p antibody (Fig. 4D). High content imaging identified mitotic cells by means of their small nuclear sizes (< 100 µm^2^) combined with their high H3S10p levels. We found a higher fraction of mitotic cells for the HaCaT cells (5.6 ± 2.2%) as compared to the HaSKpwC7 cells (4.7 ± 1.5%) (Fig. 4D), though without statistical significance.

In summary, although the HaSKpwC7 cells exhibit a significantly increased population doubling time as compared to the HaCaT cells, the two human keratinocyte cell lines show no significant differences in the cell cycle profile, S-phase distribution, or mitotic index.

### HaSKpwC7 and HaCaT have comparable plating efficiency but differ in cell density and cell motility as reflected by their desmosome connections

**Figure 5:**
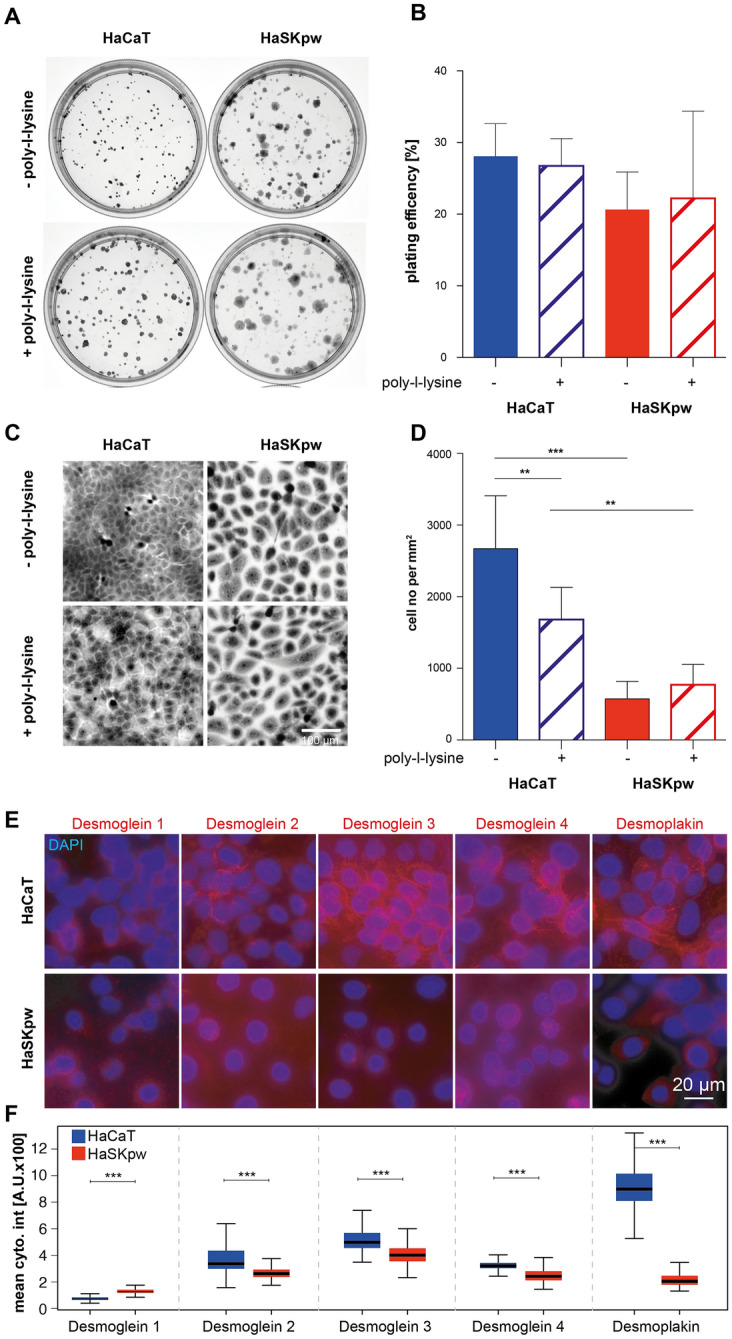
Plating efficiency and colony formation: (**A**) HaCaT and HaSKpwC7 cells were plated on regular or poly-l-lysine coated cell culture dishes, grown for 10 days and colonies were stained with Methylene-blue. (**B**) Quantification of the plating efficiency for three biological replicates (mean and standard deviation) show no significant differences. (**C**) Morphological differences of the colonies formed by HaCaT and HaSKpwC7 cells in magnified regions of the colonies. Despite some heterogeneity in cell density particularly for HaCaT cells when plated on different substrates, the HaSKpwC7 cell morphology varies strongly with apparently larger cells forming less dense cell colonies. (**D**) For quantification of the cell density, 30 randomly selected fields were analysed per condition and the cell number counted (per mm^2^). On both substrates HaCaT cells show a significantly higher cell density as compared to HaSKpwC7 cells, though poly-l-lysine coating caused significant reduction in cell density in HaCaT colonies. Statistical significance was tested with the ANOVA test, including Bonferroni post-test on a significance level of p = 0.05. (**E**) Immunofluorescent detection of desmosome components in HaCaT and HaSKpwC7 cells. Five different components of desmosomes were quantified using immunofluorescent detection combined with high content screening. Top row shows the results of the staining for Desmoglein 1–4 as well as Desmoplakin in HaCaT cells, while the lower row shows the corresponding stainings in HaSKpwC7 cells. Note that the exposure time for Desmoglein 1 was three times higher than for the other stainings. All images are represented with identical look up tables for the same staining. (**F**) Quantification of desmosomes by high content image analysis. While Desmoglein 1 is barely detectable and slightly higher in HaSKpwC7 cells all other tested Desmogleins and Desmoplakin levels are significantly higher in HaCaT cells (***p < 0.0001, Wilcoxon rank sum test). All assays were performed with HaSKpwC7 cells of passages 9–14 and HaCaT cells of passages 37–43.

Next we studied the capacity of the HaSKpwC7 cells to produce progeny by performing a plating efficiency test. Seeding 500 cells into either regular cell culture dishes or poly-l-lysine coated dishes, the HaSKpwC7 and HaCaT cells were allowed to grow into colonies for 10 days before they were stained and counted. Figure 5A shows the plating profile of the HaSKpwC7 and HaCaT cells under the different plating conditions. Plating efficiency for both cell lines on both substrates was between 20 and 30% (Fig. 5B) with no statistically significant differences between cell lines and substrates. The HaCaT colonies appeared more compact as compared to the HaSKpwC7 colonies. Quantification shows significantly more cells per mm^2^ in HaCaT colonies as compared to HaSKpwC7 colonies. Interestingly, poly-l-lysine coating reduced the cell density in the HaCaT colonies, but not in the HaSKpwC7 colonies (Fig. 5D), demonstrating that the substrate determines the anchorage of the tightly inter-connected HaCaT cells, but appears inconsequential for the less tightly inter-connected HaSKpwC7 cells.

One of the most important intercellular adhering junctions for keratinocytes are the desmosomes (e.g. Ref.^23^). Therefore, we aimed to determine the role of desmosomes in the different “compactness” of the HaCaT and HaSKpwC7 cells. Major components of this cell junctional protein complex are Desmoglein (Dsg) and Desmoplakin (Dsp)^24^. Since Ca^2+^ concentration is critical for the desmosome formation we incubated both cell lines in an equal concentration of 1.4 mM Ca^2+^ for 48 h before the amount of the desmosome proteins was assessed by quantitative immunofluorescence. While Dsg1 was barely detectable in both cell lines (Fig. 5E,F) Dsg2 Dsg3 and Dsg4 were expressed by both cell lines, though the signal levels were higher in HaCaT compared to HaSKpwC7 cells, with Dsg3 being the strongest one and showing the typical desmosomal patterns (Fig. 5E, mid top row). In contrast, Dsg4 is usually only expressed in higher levels in partly stratified HaCaT cells^25^. Similar to Dsg3, Desmoplakin 1&2 was found in HaSKpwC7 cells at a significant lower level. In HaSKpwC7 cells the desmosomal proteins are strongly reduced with a perinuclear staining for Dsg2. This corresponds well to what is seen in cSCCs, in particular also in acantholytic tumors^26,27^. A membrane-specific desmosomal pattern was only seen in HaCaT cultures. Together this strongly suggests that the presence of desmosomal proteins is strongly reduced in HaSKpwC7 cells and thus correlates well with the limited cell–cell attachment characteristic for the HaSKpwC7 cells.

**Figure 6:**
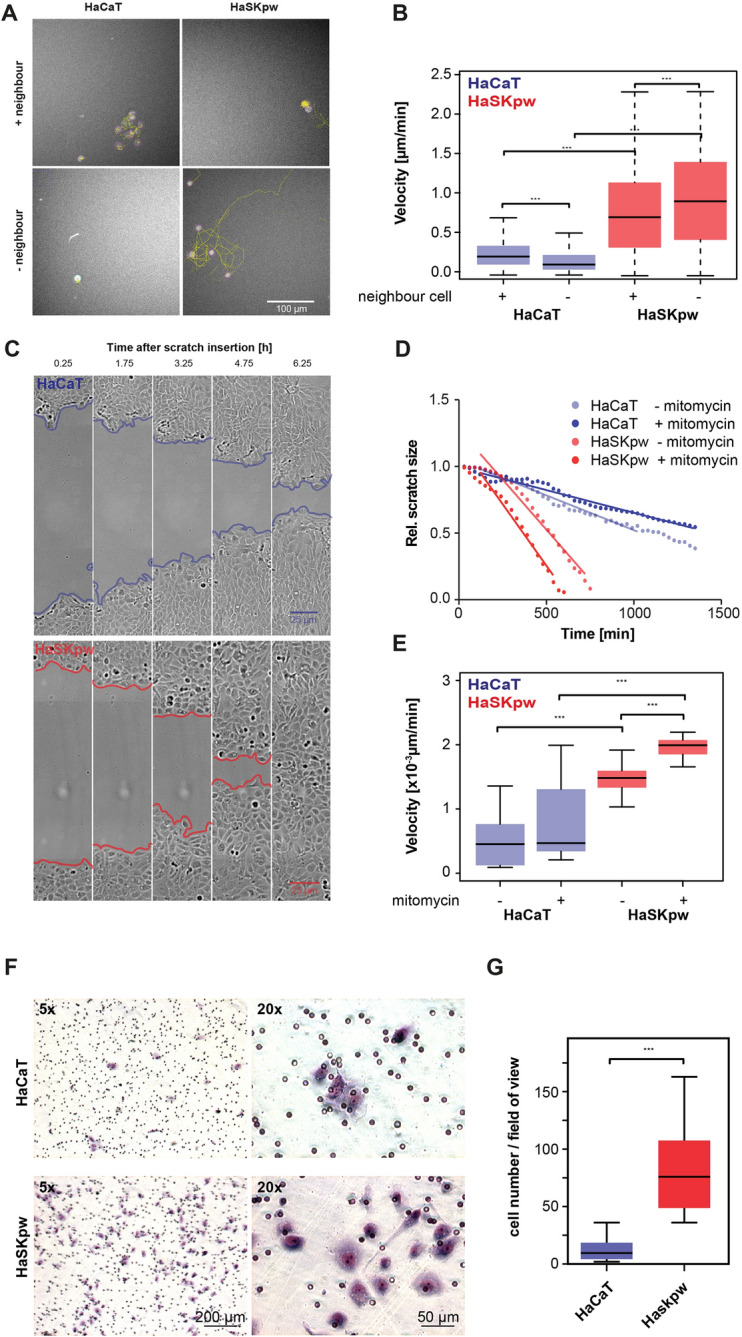
Cell migration analysis and scratch healing: (**A**) Tracking of HaCaT and HaSKpwC7 cells movement with (+) and without (−) neighbouring cells within 35 µm radius. Depicted are cells and their corresponding movement tracks for 18 h (72 frames, yellow lines) after a total of 36 h. (**B**) Boxplot of the velocity of HaCaT and HaSKpwC7 cells (n > 25 tracks) with and without neighbouring cells, indicated in µm/min). The speed was calculated from the total distance that each cell covered during the 36 h experiment. (**C**) Images of Scratch-assays performed on confluent HaCaT and HaSKpwC7 cells incubated in mytomycin C (10 µM) containing medium. Scratches were inserted and images taken at 30 min intervals with a 20 × air objective. The measured areas are marked in red for the HaSKpwC7 cells and in blue for the HaCaT cells. (**D**) Example plots for the time-dependent decrease of the relative scratch size of mitomycin C treated or untreated (dotted lines) cells with their corresponding linear regression (solid lines), calculated only for the linear parts of the graph. (**E**) Boxplot of the velocity of scratch closing calculated from linear regressions of each scratch closing analysis (n > 6). Velocity is given in µm/min. (**F**) Boyden chamber assay using dermal fibroblasts as chemoattractant in the lower chamber. HaCaT and HaSKpwC7 cells were seeded on top of a 8 µm pore membrane and incubated for 48 h prior to staining with Giemsa. On the left-hand side is a low magnification overview (×5 objective), on the right-right side a higher magnification (×20) view. While only a small number of HaCaT cells migrated through the pores, a significantly higher number of HaSKpwC7 cells was found at the bottom of the membrane. (**G**) Quantification of the Boyden chamber assay. Migrated cells were scored manually in 10 randomly selected fields per replicate using the ×5 objective magnification. Two independent replicates were analysed. Boxplots represent the 25th to 75th percentile and the centre bar represents the median. Significance was tested using t-test. All assays were performed with HaSKpwC7 cells between passages 9 and 14 and with HaCaT cells between passages 37 and 43.

The difference in attachment and intercellular connection prompted us further to analyse for the mobility of the HaSKpwC7 and HaCaT cells using the non-directed mobility assay. Living cells were stained with the far-red live cell DNA dye SiR-DNA and images were taken in 15 min intervals for several days. Cells were categorized as either “with neighbour” (+) or “without neighbour” (−) depending on the presence of another cell within a 35 µm radius (Fig. 6A). The total covered distance of individual cells was then tracked within these categories. Analyses showed that only 20% of HaSKpwC7 cells had a cell “neighbour” within a diameter of 35 µm, compared with 80% in HaCaT cultures, confirming the tight intercellular attachment of the HaCaT cells and the reduced contacts of the HaSKpwC7 cells.

We further observed that the speed of cellular movement for HaSKpwC7 cells was faster than that for HaCaT cells. Even more so, the appearance of “neighbours” influenced this speed (Fig. 6B). For HaSKpwC7 cells, having “neighbours” reduced the average speed from 0.92 µm/min (for isolated cells) to 0.76 µm/min (with another cell in close proximity). Isolated HaCaT cells moved with an average speed of 0.17 µm/min, whereas cells with “neighbours” had a velocity of 0.24 µm/min.

To explore whether this would be of functional consequence, we performed a scratch-healing assay. The scratch was introduced in confluent cultures of HaSKpwC7 and HaCaT cells and the process of gap closing was imaged every 30 min for a total of 24 h. To differentiate between cellular movement and cell proliferation, the replication inhibitor mitomycin C was added in half of the cultures (Fig. 6C). The results indicate a significantly higher scratch closing efficiency for HaSKpwC7 cell when incubated with mytomicin C (+) (1.97 × 10^–3^ µm/min) compared to untreated cells (−) with 1.49 × 10^–3^ µm/min (Fig. 6C–E). For HaCaT cells, migration efficiency was generally reduced and also the difference between mitomycin (+) and (−) was lower, with a mean velocity of 0.77 × 10^–3^ µm/min with mitomycin C treatment and 0.52 × 10^–3^ µm/min without mitomycin C, respectively. This direct comparison of the two keratinocyte lines reveals a highly significant increased scratch closing velocity for HaSKpwC7 under both conditions and thereby confirms their increased mobility (Fig. 6A,B).

As a next step, we analysed the migration properties/invasive potential of both cell lines using a Boyden chamber assay. Using conditioned medium from normal human dermal fibroblasts as chemoattractant, both cell lines were found to be able to pass through the 8 µm pores. While we found that HaCaT on average had 12.9 ± 2.4 cells per field of view, HaSKpwC7 cells showed a significant increase in migration potential with 80.1 ± 7.5 cells per field of view (Fig. 6F,G). Together, both studies underline the enhanced mobility of the HaSKpwC7 cells compared to the HaCaT cells and demonstrate that this is of functional consequence as shown for the in vitro wound healing and invasion assays.

While the Boyden chamber Assay is supposed to mimic invasive growth capacity, soft agar growth is thought to reflect the potential for tumour growth^28^. We, therefore, next assayed HaCaT and HaSKpwC7 cells for their ability to form colonies in soft agar. Interestingly, both cell lines failed to produce colonies irrespective of how many cells were plated and what agar concentration was used (data not shown). As we experienced lack of correlation for soft agar growth and tumorigenicity in nude mice for a series of carcinoma cells already previously^29^ it is tempting to suggest, that soft agar growth is not a valuable growth parameter for epidermal cells.

### HaSKpwC7 cells show differential gene expression as compared to HaCaT cells

**Figure 7:**
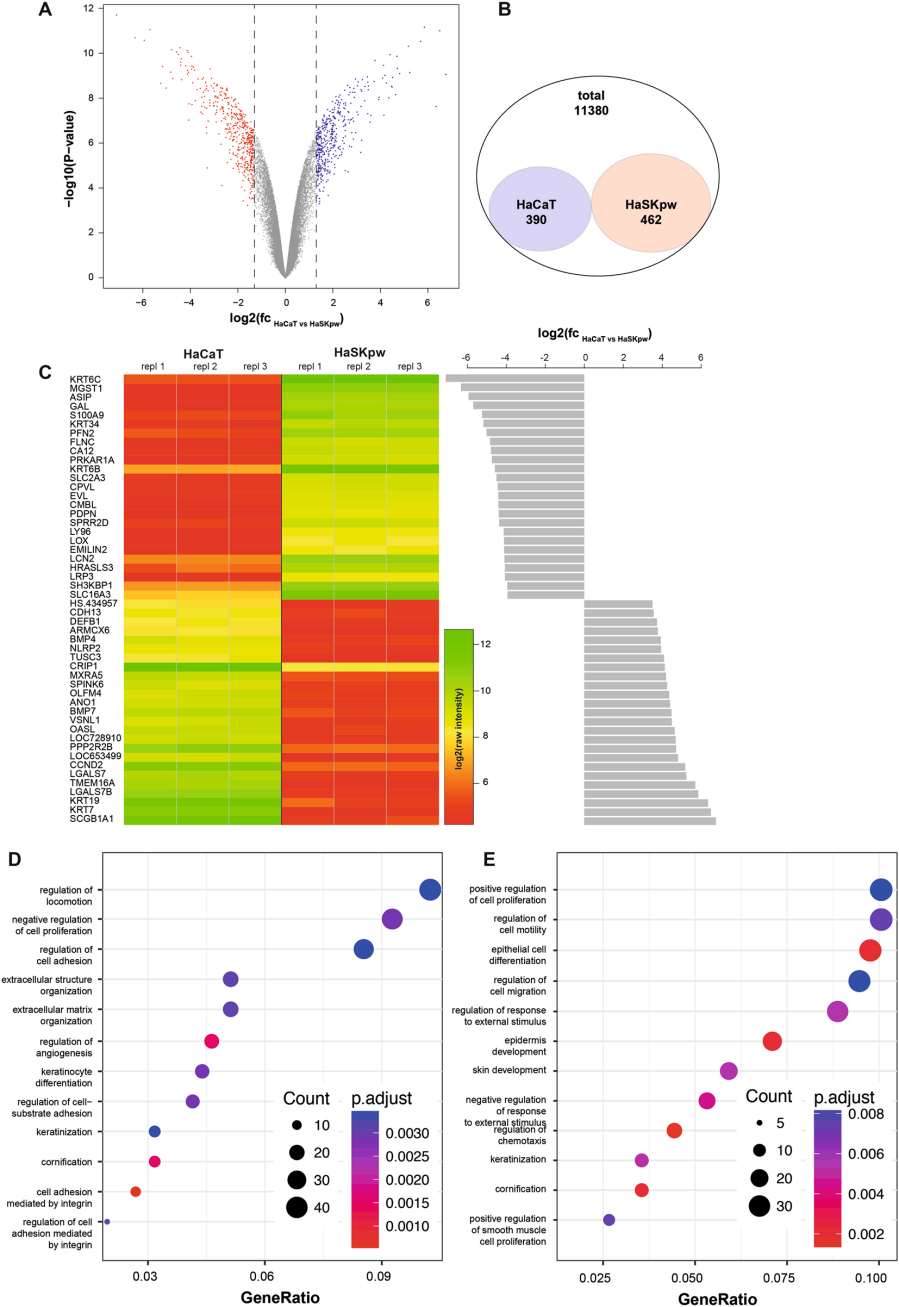
Differential expression analysis in HaCaT and HaSKpwC7 cells. (**A**) RNA expression analysis was performed and differentially expressed genes in the two cell lines HaCaT (passage 38) and HaSKpwC7 (passage 9) are represented as Volcano plot. Red dots represent significantly overexpressed genes in HaSKpwC7 cells, while blue dots represent genes overexpressed in HaCaT cells. Grey dots represent genes not significantly overexpressed (cutoff: 1.3 FC with adjusted p-values < 0.01). (**B**) Venn diagram showing the total number of significantly expressed genes and the number of genes overexpressed in HaCaT and HaSKpwC7 cells, respectively. (**C**) The heat map shows the log10 transformed raw expression values obtained for the 25 top overexpressed genes in HaSKpwC7 (top) and the top 25 overexpressed genes in HaCaT cells (bottom). The heat map depicts the raw expression levels of the genes as well as their variability. The grey bars represent the corresponding calculated fold changes after normalization. The underlying data are presented in Supplementary Table 3. Gene ontology enrichment in terms of Biological Processes of the transcripts overrepresented in HaSKpwC7 (**D**) and HaCaT cells (**E**). The clusters are sorted with increasing gene ratio, the size of the dots represents the group size, and the colour coding depicts the adjusted p-value for the enrichment calculation (p-values < 0.01 and group size > 5).

Since the HaSKpwC7 and HaCaT cells differ in their genetics and thus would likely also differ in their expression profile/molecular regulation we next compared the gene expression profile of the HaSKpwC7 and HaCaT cells by performing a differential gene expression analysis using an Illumina HumanHT-12 v4 bead array. Three biological replicates were analysed per cell line. After normalisation and validation, 11,380 transcripts were identified that were expressed above the background level in both or either one of the two cell lines. From these 11,380 transcripts 390 were overexpressed in HaCaT cells while 462 were found to be overexpressed in HaSKpwC7 cells (Fig. 7A,B). Next we selected the 25 most overexpressed transcripts (in terms of fold change and adjusted p-values) in HaCaT and HaSKpwC7 cells, respectively, and compared their expression levels within the two cell lines and the three replicates.

While the majority of genes that exhibited strong changes in expression showed a switch from low to high or vice versa (Fig. 7C), we also found significant deregulation in genes that were expressed at a medium level (yellow colour in the heatmap). Among the top deregulated genes, we found transcripts involved in epidermal generation, keratinisation, and keratin expression patterns. The complete list of differentially expressed genes is presented in Supplementary Table 2. Most notable is the over-expression of epidermal genes, KRT6B/C and KRT34^30–32^ in the HaSKpwC7 cells, while in the HaCaT cells these genes were expressed at low level. Instead the HaCaT cells over-expressed the simple epithelia keratins KRT7 and KRT19^33^. Interestingly, the LOX genes, important for keratinisation and terminal differentiation in human skin^34^ were also differentially expressed in the two cell lines showing minor expression in HaCaT cells while being over-expression in the HaSKpwC7 cells.

Next we analysed all differentially expressed genes in terms of their pathway-specific clustering using clusterProfiler^35^. The complete set of expressed transcripts was used as the gene universe. Gene ontology analyses revealed several clusters of genes related to cell proliferation, adhesion, and locomotion, thereby corresponding well to the above described differences in cell migration (Fig. 6) and growth morphology (Fig. 5). Furthermore, they explain why HaCaT cells grow more compact and with lower locomotion compared to the HaSKpwC7 cells. The second large group of differentially regulated genes falls into the cluster of epidermal development, skin formation, and cornification. This cluster appears to be over-expressed in both HaSKpwC7 and HaCaT (Fig. 7D–E), although different transcripts are involved, and nicely demonstrate their origin and tissue affiliation.

### microRNA expression in HaCaT and HaSKpwC7 show differences in terms of cell differentiation

**Figure 8:**
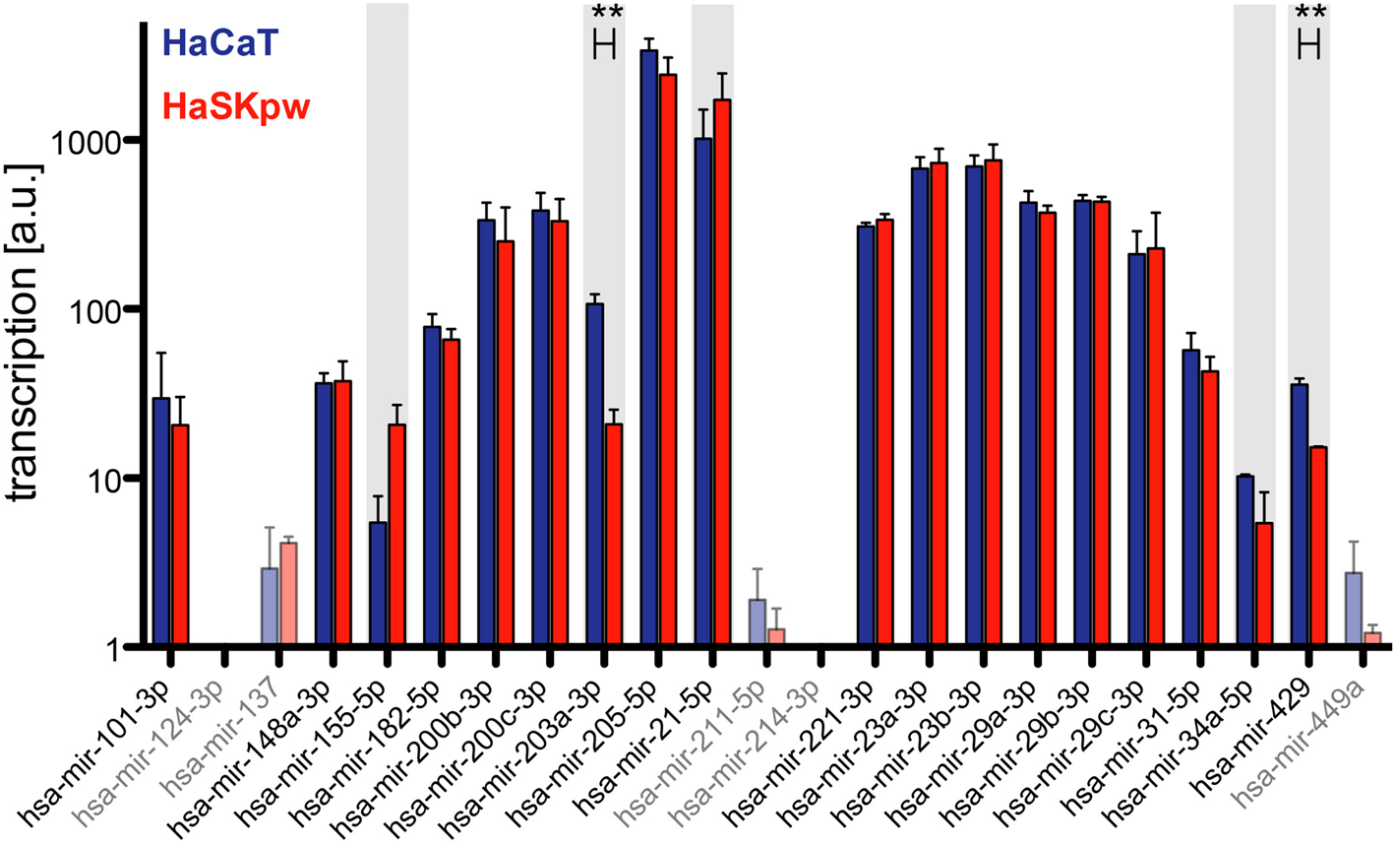
MicroRNA expression in HaCaT cells compared to HaSKpwC7 cells. Log10 raw expression values of the 23 miRNA expression levels in HaCaT (blue) and HasKpwC7 (red) cells as measured with the FirePlex assay. Five miRNAs were excluded from further analysis due to expression levels too low for robust quantification (dim bars). Five miRNAs show a differential expression with a fold change > 1.5 (grey background) two of which have a p-value < 0.01 (**; Students t-test). HaSKpwC7 cells were assayed between passage 11 and 14, HaCaT cells were analysed between passage 39 and 41.

It is well established that a number of microRNAs play a key role in the regulation of epidermal differentiation and homeostasis. To understand better the differential regulation described above we decided to analyse the transcription of 23 candidate miRNAs with potential functions in skin differentiation and skin carcinogenesis in both HaCaT and HaSKpwC7 cells via Fireplex technology (Abcam). Five miRNAs with very low expression were excluded from the analysis (presented as transparent graphs in Fig. 8). Among the remaining 18 candidates, five showed different basal transcription levels (HaSKpwC7 vs HaCaT cells). Two, hsa-miR-155-5p (+ 3.77-fold) and hsa-miR-21-5p (+ 1.7-fold) were found to be up-regulated in HaSKpwC7 cells. In contrast hsa-mir-203a-3p (− 5.16-fold), hsa-mir-34a-5p (− 1.89-fold) and hsa-mir-429 (− 2.33-fold) were down-regulated (Fig. 8).

Noteworthy is that the higher expression of hsa-miR-155-5p and hsa-miR-21-5p was also found in keloid keratinocytes^36^. Thus, for the HaSKpwC7 cells it may suggest that their increased expression is indicative of a higher oncogenic potential. Furthermore, the higher expression of hsa-mir-203a-3p, hsa-mir-34a-5p, and hsa-mir-429 in HaCaT cells is indicative for a higher differentiation potential, reduced migration and invasion as compared to the HaSKpwC7 cells^37,38^.

### HaSKpwC7 cells exhibit altered epidermal differentiation and invasive growth upon tissue reconstruction

To determine the functional consequence of the phenotypic and genotypic differences identified in vitro we investigated the HaSKpwC7 and HaCaT cells for their ability to develop and maintain an epidermis-like epithelium in 3D organotypic cultures. As we showed previously, NHEK generate skin equivalents perfectly recapitulating normal human epidermis in situ^39^. Likewise, skin carcinoma cells demonstrate their tumorigenic potential in the skin equivalents by breaking through the basement membrane and grow invasively into the underlying dermal equivalent in a manner very similar to what is seen in xenotransplants in nude mice^39^ .

**Figure 9:**
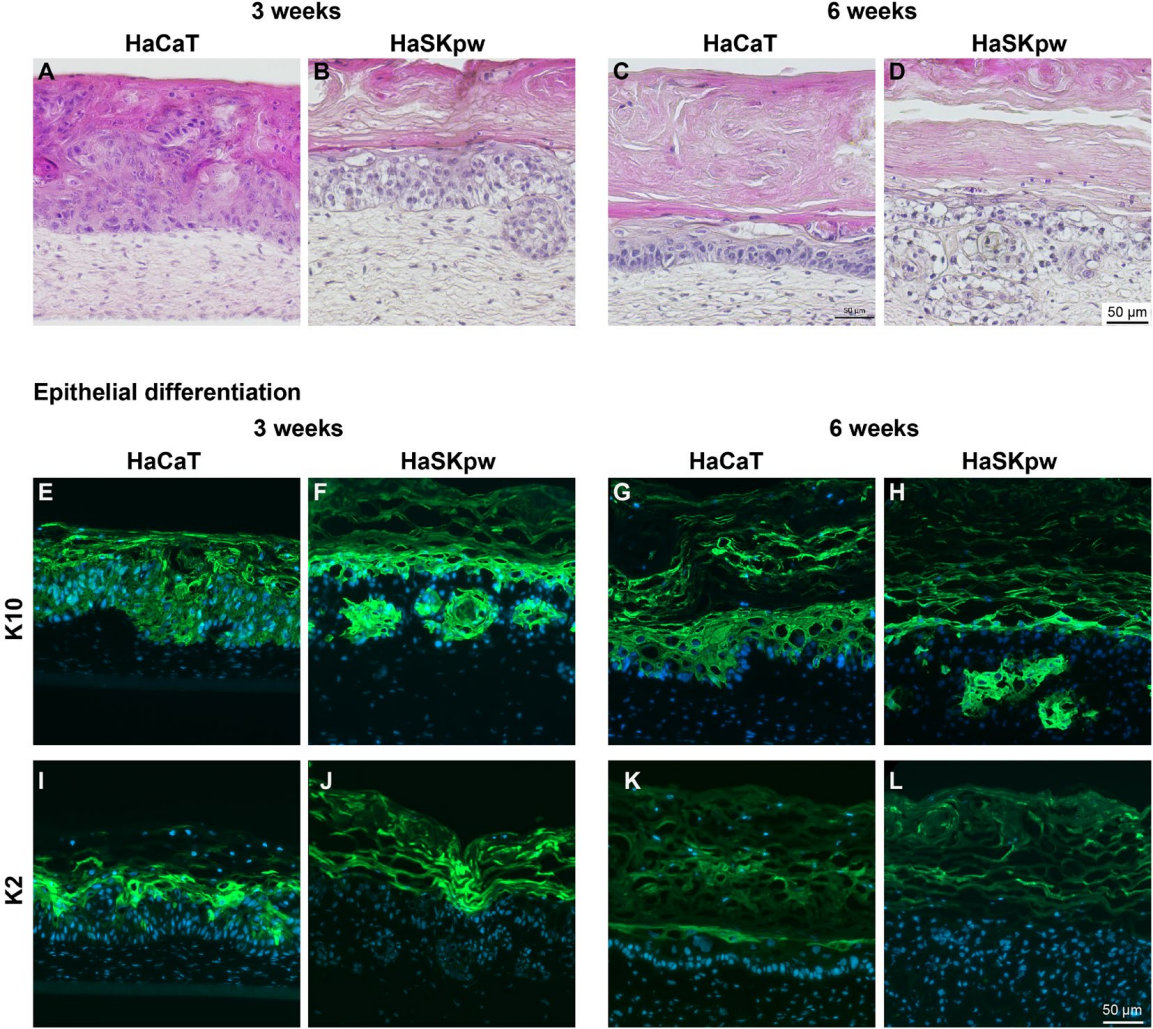
Organotypic cultures generated with HaCaT and HaSKpwC7 cells. Organotypic cultures based on a human fibroblast-derived dermal compartment (passage 8) and an either HaCaT (passage 39) or HaSKpwC7 (passage 17) cell-derived epithelium. (**A**–**D**) Histology of the HaCaT and HaSKpwC7 3-week (**A**, **B**) and 6-week-old cultures (**C**, **D**). Note, that HaSKpwC7 cells show invasive growth at both time points. (**E**–**L**). Immunofluorescence staining for differentiation-specific keratins (K10) (**E**–**H**) and K2 (**I**–**L**) both in green. Nuclear staining was done with DAPI (blue). Scale bar 50 μm.

To test the HaSKpwC7 and HaCaT cells, we produced fibroblast-derived dermal equivalents, which were complemented with HaSKpwC7 or HaCaT cells^39^. The tissues were grown for 3 and 6 weeks, respectively, before they were prepared for histological and immunohistochemical analyses (Fig. 9). Within 3 weeks, both HaSKpwC7 and HaCaT cells established a stratified, though unorganized, epithelium with a massive parakeratotic stratum corneum.

Different from the HaCaT epithelia, the HaSKpwC7 epithelia were less tightly packed and predominantly formed by large keratinocytes with abundant pale eosinophilic (glassy) cytoplasm, indicative for an altered metabolism. Most notably however, the HaSKpwC7 cells had invaded the underlying dermal equivalent. This unexpected tumorigenic phenotype proved stable also after 6 weeks (Fig. 9A–D) and presented a general trait of the HaSKpwC7 cells also further on.

The later time point was also characterized by some acantholysis. This contrasts HaCaT epithelia, which rather normalized with time, resulting in a more epidermis-like tissue organization. It was also confirmed when investigating early (Keratin 10, K10) and late (Keratin 2, K2) epidermal differentiation markers (Fig. 9E–L). While in HaCaT epithelia the expression and distribution more closely resembled that of normal human epidermis, the expression of epidermal differentiation markers was rather declined in the 6-week-old HaSKpwC7 epithelium, leaving the superficial epithelium largely undifferentiated. Interestingly, the invasive cell islands remained strongly positive for the differentiation-specific keratin 10, demonstrating that their potential for epidermal differentiation was not lost and therefore not causal for gain of invasive growth.

Thus, with the HaSKpw cells we have established a unique cell model, which immortalized spontaneously and in their early passages were used as representatives of normal human skin keratinocytes^40^—spontaneously gaining indefinite and invasive growth potential during subsequent passaging.

## Discussion

The understanding of cSCC development, its heterogeneity and progression up to metastasis is crucial for prevention and novel treatment strategies. While investigations of skin biopsies and tumour material reflect the interindividual variations of cSCC the use of immortalised cell lines as a tool to study the onset and progression of skin cancer has made it possible to truly explore different pathways involved in these processes under stable and reproducible conditions^41^. Since cell lines provide an excellent basis, we here introduced a new cell line, which has developed spontaneously from normal human adult skin, the HaSKpw cells. Particularly intriguing is, that isolated from human epidermis, early passage cells have been utilized as phenotypically normal human skin keratinocytes (NHEK), forming a perfect epidermis when used in skin equivalents and as such were applied for several stem cell studies^40^. Within this total population, immortalization occurred without crisis and with no notable selection for a specific subpopulation. The origin (Human adult skin keratinocytes) and the specific characteristic of expressing wild-type p53 protein (pw), have contributed to the name HaSKpw. To point out their characteristics in more detail we here compared a clonal population of the HaSKpw cells, HaSKpwC7, with the well-known and frequently used spontaneously immortalised HaCaT cells previously established by us^11^ and thereby highlight the uniqueness and relevance of the HaSKpwC7 cells as a new keratinocyte and skin cancer model.

Both HaSKpw and HaCaT cells have acquired several chromosomal aberrations and their fundamental genetic development appears to vary significantly. While in HaCaT cells gross chromosomal aberrations predominated (loss of entire chromosomes or chromosome arms) and polyploidization was a strong driver^11^, the genotype of the HaSKpw cells was affected quite differently. Despite being genetically highly unstable, the HaSKpw cells maintained a robust hypodiploid karyotype, which in addition to some gross chromosomal changes was characterized by highly complex translocation chromosomes.

Interestingly, chromosome 20 is one of the most affected chromosomes in the HaSKpw karyotype. This particular chromosome was already involved in aberrations in early passages (before passage 22) of the HaSKpw cells and proved to be unstable further on, forming new aberrations also seen in the clone 7 cells (HaSKpwC7). Indicated by the intrachromosomal telomeres in such translocation chromosomes, it appears that chromosome 20 predominantly associated to other chromosomes via its telomeres, suggesting for critically short, sticky telomeres rather than intrachromosomal break as a cause of rearrangements with chromosome 20. Interestingly, gain of the entire chromosome 20 is common among a number of skin cancer types^42^**.** Similarly, recurrent gain and amplification have been observed in a variety of cancers and its extensive analysis identified several cancer-related proteins for chromosome 20^43,44^. It is suggested that spontaneous 20q amplification may also cause the induction of pathway deregulation, including the MAPK- and the p53 pathway^45^. All this argues for a specific role of chromosome 20 also in the genesis of the HaSKpwC7 cell line. The 20q chromosomal arm appears of particular interest since it harbours several genes implicated in chromosomal instability^46^.

To learn more about the biological behaviour of the HaSKpwC7 cells, we studied various parameters, including their growth behaviour. We now demonstrate that although both HaCaT and the HaSKpwC7 cells start with a similar lag phase the HaSKpwC7 cells grow more slowly with a population doubling time of 43 h versus 28 h for the HaCaT cells. This has no significant effect on the cell cycle distribution compared to the HaCaT cells. As another parameter, we invesitaged the ability for clonal growth and found that although the plating efficiency of the two cell lines is similar, HaSKpwC7 cells have a significantly reduced colony density. While the HaCaT cells behave similar to normal human keratinocytes, building tight colonies with numerous cell–cell contacts (desmosomes) and the colonies are smaller in size, the HaSKpwC7 cells tend to grow more dispersed. This phenotype, which HaCaT cells acquire upon growth in serum free/low Ca^2+^ conditions, is characterised by minimal cell–cell contacts and is further substantiated by the differential expression of the desmosomal components, the Cadherins and Desmoplakins. All were under-represented in HaSKpwC7 cells compared to HaCaT cells. This corresponds well to what can be observed in cSCCs, particularly in acantholytic tumors^26,27^. Not surprisingly, this influences the speed of cellular movement, and we show that the HaSKpwC7 cell movement is significantly increased as compared to that of the tightly adherent HaCaT cells.

As far as our analysis allows, we did not find involvement of cell movement-related EMT-specific genes such as *snail*, or vimentin. However, we found a connection to actin depolymerisation and its role in cell shape modulation and mobility by the expression of KRT34 in HaSKpwC7 cells. Apparently, this cytokeratin, known for its expression in hair, is quite sensitive to actin cytoskeleton damage in mesemchymal stem/stromal cells (MSC)^47^. Furthermore, F-actin depolymerisation is known to play a role in cell deformation in keratinocytes^48^ and an increase in “softness” of the cells, promoting an invasive behaviour. In line with this, and different from the HaCaT cells, the HaSKpwC7 cells show invasive growth when propagated in 3D organotypic cultures. Of note, only tumor cells recapitulated all primary features of tumor progression, including epithelial invasion through basement membrane^39,49^, thereby confirming the relevance of the 3D organotypic cultures as an in vitro approach to characterize cancer progression. Thus, for the invasive/early tumorigenic phenotype gained by the HaSKpwC7 cells spontaneously, the connection of KRT34, F-actin and their role in invasion may well be of importance and will be the purpose of future analysis.

Associations with AK (Actinic Keratosis) and cSCC are also provided by the gene expression pattern analysis of the HaSKpwC7 cells, demonstrating overexpression of KRT6 and S100A9^50–53^. To this expression pattern we can include the overexpression of one particular epigenetic regulator, miRNA 21-5p, which has also been connected to skin tumor initiation before^54–56^.

Of particular importance, and different for the two cell lines, is the status of the p53 tumor suppressor gene. It was shown for a long time that UV-type p53 mutations are frequent and very early if not initial events in the genesis of human skin cancer^57^. Accordingly, many cells in normal human skin carry p53 mutations^58^, thereby providing a first step for genomic instability in the epidermal keratinocytes. Accordingly, the HaCaT cells, with their UV-type p53 mutations and chromosomal aberrations that are similarly characteristic for cSCCs^11,21^, proved to be an excellent model of “early-stage” skin cancer. With the HaSKpwC7 cells, we now introduce a keratinocyte model that obviously represents an alternative route of skin carcinogenesis, as these cells are devoid of p53 mutations—remain p53 wild-type—and may have gained “telomere”-dependent genomic instability. The HaSKpwC7 cells may therefore, represent a promising model to unravel p53-independent skin cancer progression.

In addition, it is noteworthy, that both cell lines established spontaneously. The beauty of spontaneous immortalization is that the cells do not face any genetic manipulation but allow for the identification of endogenous changes/aberrations—likely gained in vivo—that are possibly important for immortality and the establishment of an aberrant phenotype. While the HaCaT cells maintained many characteristics of normal human keratinocytes, the newly described HaSKpwC7 cells rapidly developed towards an invasive phenotype. To the best of our knowledge, this is the first keratinocyte line demonstrating a spontaneous initial progression to a premalignant (invasive growth) state. Thus, the new HaSKpwC7 cell line will be a valuable alternative tool in the further understanding of initiation and progression of UV-dependent skin carcinogenesis.

## Material and methods

### Cell line generation

The HaCaT cells, established and propagated as described previously^11^ were thawed from our original stock of passage 30 cells and used for the experiments in passage 35–40. The HaSKpw cell line was derived from breast skin of a normal human adult female donor. The use of donor skin was approved by the Heidelberg Ethics Commission and with written informed consent of the donor. All procedures were carried out in accordance with the relevant guidelines and regulations. The keratinocytes were isolated using standard cell isolation protocol as described previously^59,60^. In short, the epidermis was detached from the dermis by Thermolysin treatment at 4 °C overnight, followed by short-term incubation in Trypsin for 5 to 10 min at 37 °C. The single cell suspension was seeded on ɣ-irradiated (60 Gy) human fibroblast feeder cells and cultured in FAD complete medium with 10% FCS and Pen/Strep for the first passages (for media specifications see below). At later passages, cells were grown without fibroblast feeder and at passage 22, the cells were cloned using cloning rings. One of these clones, clone 7 (HaSKpwC7), was further analysed (see “Results” section). For the current experiments, HaSKpwC7 cells between passage 7 and 16 were used.

### Cell culture

HaCaT cells (Human adult low Calcium High Temperature) were cultured in DMEM (4.5 g/L Glucose, l-Glutamine, Sodium pyruvate, 3.7 g/L NaHCO_3_) (Pan Biotech) containing 10% FCS and 0.1% Pen/Strep (Pan Biotech). HaSKpwC7 cells (Human adult Skin Keratinocyte, p53 wild type) were cultured in FAD complete medium^61^ (50% DMEM Medium, 50% DMEM/F12 [(1 + 1) supplemented with 15 mM Hepes, L-glutamine) (Pan Biotech)], 0.1% Pen/Strep (Pan Biotech) and 5% FBS (Biochrom), 5 mg/L Insulin (Sigma Aldrich), 24 mg/L Adenine (Sigma Aldrich), 0.4 mg/L Hydrocortisone (Sigma Aldrich), 8.3 μg/L Cholera toxin (Sigma Aldrich) and 1 μg/L rhEGF (Thermo Scientific). For passaging, cells were detached by incubation in 0.05% EDTA/0.4% trypsin (37 °C, 5 min) (Pan Biotech) and re-plating at a density of 5,600 cells/cm^2^.

Normal Human skin fibroblasts were isolated from explant cultures of normal human skin samples and routinely cultured in DMEM based medium containing 10% FBS and 0.1% Pen/Strep^39^. Passage 7–9 cells were used for generating the dermal equivalents. When used as feeder cells fibroblasts were γ-irradiated (60 Gy) and seeded at a density of 2,800 cells/cm^2^ in FAD complete media.

### Growth curve, cell cycle analysis, S-phase detection and mitotic index

10,000 cells of either HaCaT or HaSKpwC7 keratinocytes were seeded in their corresponding complete medium. For each cell line four replicates were set up and the cell number was counted every 24 h for 8 days. The resulting cell numbers were averaged and normalized to the initially seeded cell number and fitted using an exponential growth function (y = y_0_ × exp(k × x)). The cell doubling times were calculated from the fitted parameters.

Cell cycle analysis was performed using a S3 (Biorad) cell sorter, following quantitative DNA staining with propidium iodide according to Ref.^62^. In short, exponentially growing cells were trypsinised, fixed in cold EtOH for 15 min, and stained in PI staining buffer (0.1% Triton X100, 0.2 mg/mL RNase A, 20 µg/mL PI in PBS) for 30 min at room temperature. After filtering through cell-strainer-cap tubes a minimum of 15,000 gated cells were analysed. For S-phase detection cells were grown on coverslips overnight, before 10 µM EdU (Invitrogen) was added to the medium and incubated for 30 min. Then cells were fixed in 3.7% formaldehyde and washed three times in PBS/0.05% Tween 20. Cells were permeabilized in 0.5% TritonX 100 in PBS for 20 min and incorporated EdU was detected by the ClickIT Edu detection kit (Invitrogen) using 0.1 µL 6-Fam Azide per 25 µL reaction. The reaction was performed at room temperature for 45 min. After incubation, coverslips were again washed in PBS-Tween three times and DNA was stained with DAPI (1 µg/mL in H_2_O) for 10 min. Finally, coverslips were mounted in Mowiol and imaged using an Operetta High Content Imager (Perkin Elmer) with a 20 × air objective. One hundred fields of view were analysed per coverslip. Image analysis was performed using Harmony software (Perkin Elmer, version 3.5, https://www.perkinelmer.com/uk/product/harmony-4-9-office-license-hh17000010). Nuclei were identified and the corresponding intensities for the DAPI and FITC channel were recorded. S-phase cells were identified as the population with high EdU intensity and sub G2/M DNA content by its integrated DAPI intensity. For measuring the mitotic index, cells were seeded and fixed as described above. Mitotic cells were identified after staining the cells with a rabbit anti-H3S10-phospho antibody (clone MC463, Millipore, 1:200) and a goat anti-rabbit-IgG labelled with Alexa fluor 594 (Jackson Immunolabs, 1:500). DNA was counterstained with DAPI (see above). Imaging and image analysis were done as described above. Mitotic cells were identified by their high H3S10p intensity, small area and high mean DAPI intensity.

### Plating efficiency and colony formation

HaCaT and HaSKpwC7 cells were trypsinised and single cell suspensions were counted. 500 cells per experiment were plated on normal tissue culture dishes (60 mm) or on poly-l-lysine coated tissue culture dishes (1 mg/mL per 25 cm^2^ surface area). Cells were grown for 10 days, fixed in ice cold MeOH and stained with Methylene blue solution (0.2% in 50% MeOH, 50% PBS) before rinsing in ddH_2_O. All seeding experiments were performed in triplicates.

### Mobility assay

HaCaT and HaSKpwC7 cells were seeded at a density of 250 cells/cm^2^ in 12 well plates (Sarstedt) in their appropriate culture medium, supplemented with 500 nM SiR DNA stain (Spirochrome), for 4 h. Then, cells were washed twice to remove dead cells and fresh medium (including 500 nM SiR DNA) was added. Cells were imaged using an Operetta high content imager equipped with a live cell environment (Perkin Elmer). Brightfield and Cy5 (ex: 652/em: 674 nm) channels were recorded every 15 min for 36 h using a 20 × air objective. 24 fields were imaged per sample. Images were analysed using imageJ and the Track Mate^63^ plugin with the following settings: Estimated Blob Diameter: 15 µm, Threshold: 80, Filters on spots: Quality 174, Estimated Diameter 8.72, Settings for simple LAP tracker: Linking max distance 35 µm, Gap-closing max distance 35 µm, Gap-closing max frame gap: 3.

### Boyden Chamber assay

A Boyden assay^64^ was performed for HaCaT and HaskpwC7 cells in the presence of human fibroblast MSU 1–1 cells. MSU 1–1 cells were seeded in DMEM 10% FBS in 6-well plate (lower compartment of the chamber) at a density of 1 × 10^5^/mL and cultured for 24 h. The Boyden chamber inserts corresponding to the upper compartment, (23.1 mm diameter, 8 μm porous PET membrane, Falcon, Corning, NY, USA) were pre-coated with 10 μg/mL rat tail collagen I (Gibco, Life technologies, CA, USA) according to supplier's protocol, air-dried overnight, and rehydrated with complete FAD medium for 1 h before usage. HaCaT and HaSKpwC7 cells (2 × 10^5^ cells/well) were seeded on the apical surface of the insert in FAD medium without FBS and supplements. After 1 h these inserts were transferred into wells containing fibroblasts and cultured for 48 h. Subsequently, cells on the lower side of the membrane were washed three times with PBS, fixed with 3.7% paraformaldehyde for 15 min, washed twice with PBS, permeabilized with methanol for 10 min, air-dried, and finally stained with 10% Giemsa in ddH_2_O solution for 1.5 h. After staining the cells from the upper chamber (apical part of insert) were removed and cells that had trans-migrated the membrane were counted. For each cell line the test was performed in two biological replicates, and 10 fields per sample were analysed at 5 × magnification.

### Scratch assay

In order to distinguish between proliferation-dependent and migration-induced scratch healing, the scratch assay was performed in the presence and absence of mitomycin C. HaCaT and HaSKpwC7 cells were grown to confluence on 8-well chambered cover glass (Sarstedt). One h before the scratched was induced half of the wells were supplemented with mitomycin C (final concentration of 10 µM, Sigma Aldrich), while the other wells were kept in medium without mytomycin C. The scratch was set by using a fine pipet tip. Then the cells were transferred to the Operetta high content imager and phase contrast images were taken with a 20 × air objective every 15 min for a total of 48 h. Analysis of the scratch closing was done using imageJ and the plugin “Mitobo Scratch Assay”^65^.

### Desmosome staining

HaCaT and HaSKpwC7 cells were grown until confluency on cover glass. HaCaT cultures were incubated in regular medium supplemented with Ca^2+^ to a final concentration (1.4 mM) equal to the Ca^2+^ concentration in HaSKpw medium, since it was shown that Ca^2+^ concentration affects the formation of desmosomes^66^. After 48 h of incubation cells were fixed in − 20 °C MeOH for 3 min, followed by 30 s in − 20 °C cold Aceton. Cells were permeabilized in 0.1% Triton X/PBS for 5 min, followed by blocking in 3% BSA/PBS for 20 min at RT. The following primary antibodies were used: mouse-anti-Desmoglein 1 (clone Dsg1-P124), mouse-anti-Desmoglein 2 (clone G129), mouse-anti-Desmoglein 3 (clone Dsg-G194), guinea pig-anti-Desmoglein 4 (#GP4S) and mouse-anti-Desmoplakin 1&2 (clone DP2.15/2 17/2.20) all from Progen, used undiluted. Slides were incubated for 1 h at RT with primary antibodies, washed 3 times in PBS and shortly in ddH_2_O followed by a detection with secondary antibodies (Cy3-conjugated donkey-anti-mouse-IgG (Jackson Immunoresearch) 1:800 or Cy3-conjugated donkey-anti-guinea pig IgG (Dianova) 1:300) for 1 h at RT. After 3 washes in PBS slides were counterstained and mounted in Vectashield. Imaging was done using the Operetta high content imager (Perkin Elmer) using a 20 × air objective and filters for DAPI and Cy3. Image analysis was performed using the Harmony software (Perkin Elmer, version 3.5, https://www.perkinelmer.com/uk/product/harmony-4-9-office-license-hh17000010) with nuclear and cytoplasm segmentation. A minimum of 5,000 cells per staining were analysed.

### p53 sequencing

Genomic DNA from HaCaT and HaSKpwC7 cells was isolated using DNeasy Blood & Tissue Kit and total RNA was isolated using RNeasy (both Qiagen) according to the manufacturer’s instructions. All coding regions of the p53 gene locus were amplified using the primers listed in Supplementary Table S4 and the Q5 proofreading polymerase system (NEB).

Subsequently the amplicons were cloned into pMiniT 2.0 (NEB) and positive clones were sequenced using T7 and Sp6 primers. Total RNA was reverse transcribed using M-MuLV Reverse Transcriptase (NEB) and p53 mRNA was amplified using the primers given in Supplementary Table S4 and the Q5 proofreading polymerase system. The resulting DNA fragment was cloned and sequenced as described above.

The resulting sequences were aligned to the hg38 reference genome or NM_000546 respectively.

### p53 Western blot

HaCaT and HaSKpwC7 cells were harvested and lysed in RIPA buffer. Protein concentration was determined by 660 nm Protein Assay (Pierce). Indicated amounts were loaded. After separation on a 12% PAA-SDS gel, the bands were visualized using mouse-anti-actin (clone AC-40, 1:2,000) and rabbit-anti-p53 (Cell signalling, #9282, 1:1,000) followed by anti-mouse-IgG-Cy3 and anti-rabbit-IgG-Cy5 (both Jackson ImmunoResearch, 1:3,000).

### Telomerase (TRAP) assays

Cell lysis of cultured cells and the telomerase assay were performed using the TRAPeze kit (Intergen Company, Oxford, UK) as described in detail previously^15^. In brief, protein was isolated from the different samples and 50 ng of each sample were used for separation in non-denaturing 10% polyacrylamide gels. RNase-inactivated samples (RNase, DNase-free, Roche) were included as controls. Fragment distribution was visualized by auto-radiography and PhosphorImager scanning (Fujifilm Bas-1500). TINA 2.0 software was used for quantification.

### hTERT splice variant analysis

To detect differently spliced isoforms of hTERT, mRNA was isolated from HaSKpwC7 and HaCaT cells at the indicated passages. Splicing isoforms were detected according to Refs.^16,67^. In brief, total RNA was isolated using RNeasy (QIAGEN). cDNA was generated from 1 μg of total RNA (Omniscript, QIAGEN). Four μL were amplified in a 25 μL mixture of 0.2 mM dNTPs (Roche), 2.5 units Taq (Roche), and 0.2 μM of each primer, TERT-HT2026F and TERT-HT2482R (see Supplementary Table S4)^68^ for 35 cycles of 94 °C/15 s, 60 °C/15 s, and 72 °C/30 s. GAPDH was amplified with GAPDH-fw and -rv primers as internal control for 23 cycles of 94 °C/30 s, 60 °C/30 s, and 72 °C/60 s. PCR products were separated in 2% agarose (FMC Bioproducts) gels.

### Telomere length measurement

DNA from HaCaT and HaSKpwC7 cells was extracted at the indicated passages using DNA Mini Kit (Qiagen). Telomere length was quantified by quantitative polymerase chain reaction (qPCR) as previously described^69,70^. Serial tenfold dilutions of telomere standard (TEL STD) and single copy gene (SCG) standard for acidic ribosomal phosphoprotein (36B4 STD) were used to obtain a standard curve. Primers used are listed in Supplementary Table S4.

Analysis was performed using an ABI StepOnePlus Real-Time PCR System. Master mix for a 20 μL reaction was prepared with 10 μL Platinum™ SYBR™ Green qPCR SuperMix-UDG w/ROX (Thermo Fisher), 20 ng of sample gDNA and 0.1 µM of each primer specific to telomeres (T) or single copy gene (S) Each standard sample was kept at a constant DNA concentration of 20 ng per reaction by addition of plasmid DNA (pBR 322). Data analysis with normalisation to single copy gene numbers and number of chromosomes was performed according to Ref.^70^.

### Karyotype of cell lines and STR verification

M-FISH analysis was performed as described in detail elsewhere^71^. Short Tandem Repeat (STR) profiling of HaSKpw cells was done for three different passages by the DSMZ (Deutsche Sammlung von Mikroorganismen und Zellkulturen GmbH) according to Ref.^72^ and reported in Ref.^73^.

### Expression analysis

Total RNA was extracted from confluent HaCaT and HaSKpwC7 cell lines using RNeasy (Qiagen) following the manufacturer's instructions. Quality was controlled using a total RNA Nano chip assay on an Agilent 2100 Bioanalyzer (Agilent Technologies GmbH, Berlin, Germany). Three independent replicates per cell line were processed and Biotin-labelled cRNA samples for hybridization were prepared according to Illumina's recommended sample labelling procedure based on the modified Eberwine protocol^74^. In brief, 200 ng total RNA was used for complementary DNA (cDNA) synthesis, followed by an amplification/labelling step (in vitro transcription) to synthesize biotin-labelled cRNA (Roche Applied Science, Penzberg, Germany) according to the Illumina Total Prep RNA Amplification Kit (Life Technologies). The cRNA was column purified according to TotalPrep RNA Amplification Kit, and eluted in 60 µL of water. Quality of cRNA was controlled by the RNA Nano Chip Assay on an Agilent 2100 Bioanalyzer and spectrophotometrically quantified (NanoDrop). Ten microliters of labelled RNA (150 ng/µL) were hybridized to the HumanHT-12 v4 bead array, at 58 °C for 20 h, spike-in and mismatch controls were added. Post hybridisation arrays were washed in High Temp Wash buffer (Illumina Inc.) at 55 °C C and then twice in E1BC buffer at room temperature. Array signals were developed by a 10-min incubation in 2 mL of 1 µg/mL Cy3-streptavidin (Amersham Biosciences, Buckinghamshire, UK) solution and Block E1 buffer. After a final wash in E1BC, the arrays were dried and scanned. Raw data were obtained using a HiScan system (Illumina). Data extraction was done for all beads individually, and outliers were removed when > 2.5 MAD (median absolute deviation). Quality control was performed using Genome Studio (Illumina). Further processing steps were carried out using Bioconductor with the following packages GOstats, GenomicRanges, Biostrings, beadarray, limma, GEOquery, illuminaHumanv4.db, topGO, org.Hs.eg.db and clusterProfiler. The data was deposited at GEO (https://www.ncbi.nlm.nih.gov/geo/) under the accession number GSE143521.

### miRNA expression analysis

miRNA was isolated from HaCaT and HaSKpwC7 cells using miRNeasy mini kit (Qiaqen). The transcription of miRNAs was measured via flowcytometric quantification of barcode-labelled fluorescent miRNA-hydrogel-microparticle (“FirePlex Particle Technology “, Abcam) according to the manufacturer’s protocol. Briefly, one µg of RNA was added to customized firefly particles (~ 35 µL) and incubated under shaking (~ 750 rpm) at 37 °C for 90 min. After binding of miRNAs, the particles, which contain complementary sequence were rinsed with rinse buffer twice and followed by a labelling reaction (RT, 45 min, 750 rpm). During labelling each miR is ligated to two linkers. After washing a fluorescent reporter was added (RT, 45 min, 750 rpm) that binds to the miR-linker-complex. Fluorescence of the particles was then measured by flow cytometry (with e.g. Guava easycyte 8HT, Millipore). The raw data obtained from flow cytometry were then processed with the “FirePlex Analysis Workbench software” (Abcam). For normalization the geometric mean of transcription of RNU6B, RNU44 and RNU48 was determined. A miRNA is regarded as differentially expressed when the fold change is ≥ 1.5.

### Formation of organotypic cultures

Organotypic cultures were prepared according to the protocol published in Refs.^39,75^. In brief, a human fibroblast derived matrix was established and co-cultures were constructed with either HaCaT or HaSKpwC7 cells on top of the dermal compartment.

#### Human fibroblast derived matrix

Normal Human dermal fibroblasts (NHDF) of passage 5 were expanded over a maximum of 3 passages. A total amount of 1.5 × 10^6^ NHDFs was seeded onto one 12-well ThinCert with high-density 0.4 μm pores (Greiner Bio-One, 665640) placed in deep-well plates (Greiner Bio-One, 665110) and incubated at 37 °C, 5% CO_2_. For the experiments performed herein, the fibroblasts were seeded in a step-wise fashion with 3 times 0.5 × 10^6^ cells every second day. These cultures were kept submerged for 3 weeks in fdmDE-media consisting of 1:1 DMEM and DMEM/Ham’s F12 (Pan Biotech), supplemented with 10% FCS, 1% antibiotics/antimycotic (Pen/Strep/Amphotericin B Mix, Pan Biotech), 200 μg/mL 2-phospho-l-ascorbic acid-trisodium salt (Sigma), 1 ng/mL human recombinant Transforming Growth Factor-β (Life technologies), 2.5 ng/mL human recombinant Epidermal Growth Factor (EGF, Life technologies), 5 ng/mL Basic Fibroblast Growth Factor (bFGF, Life technologies), and 5 μg/mL human recombinant insulin (Sigma). The medium was exchanged every second day.

#### Skin equivalents

Twenty-four hours before seeding the epithelial cells on top of the dermal equivalent the medium was changed to rFAD media consisting of 1:1 DMEM and DMEM/Ham’s F12 (Pan Biotech), 10% FCS and 1% antibiotics/antimycotic (Pen/Strep/Amphotericin B Mix, Pan Biotech), 0.4 μg/mL hydrocortisone (Sigma) and 1 × 10^–10^ M choleratoxin (Sigma) as described^75^. The medium in the upper well was removed and replaced with 1 mL suspension of 0.25 × 10^6^ cells/mL of either HaCaT or HaSKpwC7 cells. After 14–16 h the cultures were raised to the air–liquid interface by restricting the medium level to the lower part of the chamber. The cultures were kept in rFAD media for 3 and 6 weeks and harvested for both cryopreservation (using Tissue Tec, Weckert Labortechnik) and paraffin embedding.

### Tissue differentiation in organotypic cultures

Indirect immunofluorescence was performed for determining the differentiation potential of the tissues formed in organotypic cultures. Seven µm cryosections were prepared and underwent fixation in methanol (80%, 4 °C, 10–5 min in case of Keratin 2) and subsequently acetone (100%, − 20 °C, 2 min), followed by a blocking step with 3% (w/v) BSA (in PBS) for 30 min. For Keratin10 detection (Progen, GP-K10, 1:500), primary antibody incubation occurred overnight at 4 °C and secondary at RT for 1 h (Dianova, 1:200). For Keratin 2 detection (Progen, 65191, 1:200) primary antibody binding required 30 min incubation at 37 °C followed by a second incubation at 4 °C overnight. The secondary antibody (Dianova, 1:200) was also provided in a two-step fashion with a first incubation for 30 min at 37 °C and a second one for 45 min at RT. All antibodies were diluted in blocking solution. Stained sections were mounted with coverslips using DAPI containing Vectashield Mounting Media (Biozol). Samples were examined with an Imager M2 microscope (Zeiss) equipped with an AxioCam MRm (Zeiss).

## Supplementary information

Supplementary file1

Supplementary file2

## Data Availability

The datasets generated during and/or analysed during the current study are available in the GEO repository under the accession number GSE143521. The HaSKpwC7 cells are available from Petra Boukamp upon request.
