# Supplementary material for: Characterisation of the novel spontaneously immortalized and invasively growing human skin keratinocyte line HaSKpw
Source: Sci Rep. 2020 Sep 16;10:15196. doi: 10.1038/s41598-020-71315-0 (PMC7494900; doi:10.1038/s41598-020-71315-0)
Supplement: Supplementary file 1 — Supplementary file1 [file 41598_2020_71315_MOESM1_ESM.pdf]

Characterisation of the novel spontaneously immortalized and invasively growing human skin keratinocyte line HaSKpw

Elizabeth Pavez Lorie[1*)], Nicola Stricker[2*)], Beata Plitta-Michalak[2*)], I-Peng Chen[3)], Beate Volkmer[3)], Rüdiger Greinert[3)], Anna Jauch[4)], Petra Boukamp[1#)] and Alexander Rapp[2#)]

Supplementary Information

Supplementary Tables

Table S1: Detected differences between the reference sequence for p53 and the assembled amplicons. Alignment positions based on NC_000017, Homo sapiens chromosome 17, GRCh38.p12 primary assembly.

Nt position	Location	HaSKpw	HaCaT
11821	Intron 1		T→A
13032	Intron 4	T→C	T→C
13140	Intron 4	T→-	T→-
13141	Intron 4	T→-	T→-
13142	Intron 4	T→-	T→-
13190	Intron 4	T→C	
13224	Intron 4	G→A	G→A
13259	Intron 4	T→a	
13474	Exon 5		C→t
13754	Intron 5		A→G
14011	Intron 5		A→t
14058	Intron 5		C→t
14146	Intron5		G→t
14774	Exon 8		C→t
14775	Exon 8		C→t
14972	Exon 9	C→t	
15406	Intron 10	insert aaa	
15521	Intron 10	G→a	
15593	Intron 10	A→t	
15721	Intron 10		C→g
15867	Intron 10	C→a	
19617	Exon 11		C→t
19697	Exon 11		T→-
19698	Exon 11		T→-
19763	Exon 11	G→a	
19768	Exon 11	G→a	

Table S2: Table of differentially expressed transcripts between HaCaT and HaSKpw cells. Table S2 is provided as a separate Excel file. The file summarizes the differential expressed transcripts detected by the expression analysis.

Table S3: 25 most up-regulated and 25 most down-regulated transcripts between HaCaT and HaSKpwC7 cells. Fold change is expressed as logFC, the average expression and the t represents the absolute t-statistic, the raw and adjusted p-values and the B-statistic as calculated using Limma.

TargetID	logFC	AveExpr	t	P.Value	adj.P.Val	B
KRT6C	-7.107735	9.063761	-94.426114	2.01E-12	6.92E-08	17.224219
MGST1	-6.329242	7.613881	-68.328189	2.07E-11	1.43E-07	16.050764
ASIP	-5.940545	7.267654	-65.933711	2.67E-11	1.47E-07	15.895421
GAL	-5.698182	7.230993	-76.836639	8.87E-12	8.66E-08	16.525212
S100A9	-5.247698	7.827064	-35.763897	2.18E-09	1.32E-06	12.496452
KRT34	-5.168909	7.141347	-45.362839	3.95E-10	5.44E-07	13.968798
PFN2	-5.016641	7.829922	-33.395239	3.56E-09	1.65E-06	12.04351
FLNC	-4.83476	6.858679	-41.928486	6.95E-10	7.33E-07	13.499867
CA12	-4.789329	7.043763	-57.618779	7.06E-11	2.35E-07	15.262214
PRKAR1A	-4.740886	6.85457	-38.299604	1.33E-09	1.04E-06	12.937322
KRT6B	-4.584614	9.302626	-55.783832	8.91E-11	2.41E-07	15.09954
SLC2A3	-4.506266	6.984198	-53.924162	1.14E-10	2.64E-07	14.924823
CPVL	-4.443161	6.933238	-59.411087	5.66E-11	2.17E-07	15.412435
EVL	-4.412164	6.795556	-46.339108	3.39E-10	5.41E-07	14.092171
CMBL	-4.407974	6.69283	-48.369492	2.49E-10	4.29E-07	14.335975
PDPN	-4.382756	6.524495	-50.690732	1.77E-10	3.40E-07	14.595127
SPRR2D	-4.36733	7.130543	-37.660176	1.50E-09	1.06E-06	12.830125
LY96	-4.13353	6.453905	-57.13651	7.50E-11	2.35E-07	15.220356
LOX	-4.118901	6.341101	-40.876669	8.34E-10	7.99E-07	13.344418
EMILIN2	-4.114423	6.429674	-43.038719	5.76E-10	6.40E-07	13.657714
LCN2	-4.095406	8.519775	-55.614711	9.11E-11	2.41E-07	15.084071
HRASLS3	-4.073778	7.88604	-21.097911	9.49E-08	1.16E-05	8.777889
LRP3	-4.071233	6.443946	-41.869629	7.02E-10	7.33E-07	13.491323
SH3KBP1	-3.95293	8.705902	-52.551718	1.37E-10	2.78E-07	14.789079
SLC16A3	-3.941327	9.546908	-43.743601	5.12E-10	6.09E-07	13.754752
HS.434957	3.496649	6.258125	34.394396	2.88E-09	1.52E-06	12.239761
CDH13	3.562402	6.711933	20.479732	1.17E-07	1.35E-05	8.556637
DEFB1	3.73432	6.513196	36.731828	1.80E-09	1.24E-06	12.669798
ARMCX6	3.772453	6.207947	49.540192	2.09E-10	3.80E-07	14.469169
BMP4	3.924585	6.920986	28.366331	1.15E-08	3.18E-06	10.922984
NLRP2	3.942632	6.62426	29.242791	9.23E-09	2.81E-06	11.135921
TUSC3	4.090142	6.410093	38.553575	1.27E-09	1.04E-06	12.979189
CRIP1	4.130876	10.257815	46.13441	3.49E-10	5.41E-07	14.066644
MXRA5	4.189577	7.535678	39.997559	9.75E-10	8.85E-07	13.209864
SPINK6	4.255031	7.057783	35.914326	2.11E-09	1.32E-06	12.52382
OLFM4	4.355178	7.076055	29.030141	9.72E-09	2.84E-06	11.085005
ANO1	4.404136	7.065588	53.862589	1.15E-10	2.64E-07	14.91886
BMP7	4.472928	7.493898	34.239942	2.98E-09	1.53E-06	12.209933
VSNL1	4.491968	6.969908	33.913675	3.19E-09	1.59E-06	12.146317
OASL	4.639255	7.214491	30.420785	6.95E-09	2.37E-06	11.409667
LOC728910	4.694157	6.962395	52.577897	1.36E-10	2.78E-07	14.791724
PPP2R2B	4.710264	8.352951	44.274621	4.70E-10	6.00E-07	13.826283
LOC653499	4.813132	6.873365	41.100314	8.02E-10	7.90E-07	13.377967
CCND2	5.17793	8.458227	60.577148	4.92E-11	2.12E-07	15.505841
LGALS7	5.235535	7.397774	41.650015	7.29E-10	7.39E-07	13.459284
TMEM16A	5.696273	7.321595	64.905482	2.99E-11	1.47E-07	15.825337
LGALS7B	5.85032	7.704137	79.50071	6.94E-12	8.66E-08	16.65249
KRT19	6.348186	8.516244	25.686267	2.33E-08	4.90E-06	10.217462
KRT7	6.498728	7.78835	75.517788	1.01E-11	8.66E-08	16.458753
SCGB1A1	6.7548	8.374901	40.541224	8.85E-10	8.25E-07	13.293584

Table S4: Oligonucleotide Sequences

Primer	Sequence
p53 sequencing	
p53.a1.fw	5'-TCTCAGACACTGGCATGGTG;
p53.a1.rv	5'-TTGGCAAAACATCTTGTTGAGG
p53.a2.fw	5'-TTCACTTGTGCCCTGACTTTC
p53.a2.rv	5'-CCTCCACCGCTTCTTGTCC
p53.a3.fw	5'-TAATCTACTGGGACGGAACAGC
p53.a3.rv	5'-GCGACAGAGTGAGACTGAGTC
p53.a5.fw	5'-AGGCGGAGATTGCAATCAGC
p53.a5.rv	5'-AGTCCTGGGTGCTTCTGACG
p53.a6.fw	5'-ACATTCTCCACTTCTTGTTCCC
p53.a6.rv	5'-CAGACTCAGGTGGCTGCTTCC
p53mRNA.fw	5'-AAGTCTAGAGCCACCGTCCA
p53mRNA.rv	5'-TGGGGGTGGGAGGCTGTCAG
hTert isoforms	
TERT-HT2026F	5'-GCCTGAGCTGTACTTTGTCAA
TERT-HT2482R	5'-CGCAAACAGCTTGTTCTCCATGTC
GAPDH.fw	5'-GAGAAGGCTGGGGCTCATTT
GAPDH.rv	5'-CAGTGGGGACACGGAAGG
Telomer length	
TEL-STD	5'-$(TTAGGG)_{14}$
36B4-STD	5'-CAGCAAGTGGGAAGGTGTAATCCGTCTCCACAGACAAG GCCAGGACTCGTTTGTACCCGTTGATGATAGAATGGG
eloF	5'-CGGTTTGTTTGGGTTTGGGTTTGGGTTTGGGTTTGGGTT
teloR	5'-GGCTTGCCTTACCCTTACCCTTACCCTTACCCTTACCCT
36B4F	5'-CAGCAAGTGGGAAGGTGTAATCC
36B4R	5'-CCCATTCTATCATCAACGGGTACAA

Supplementary Figures

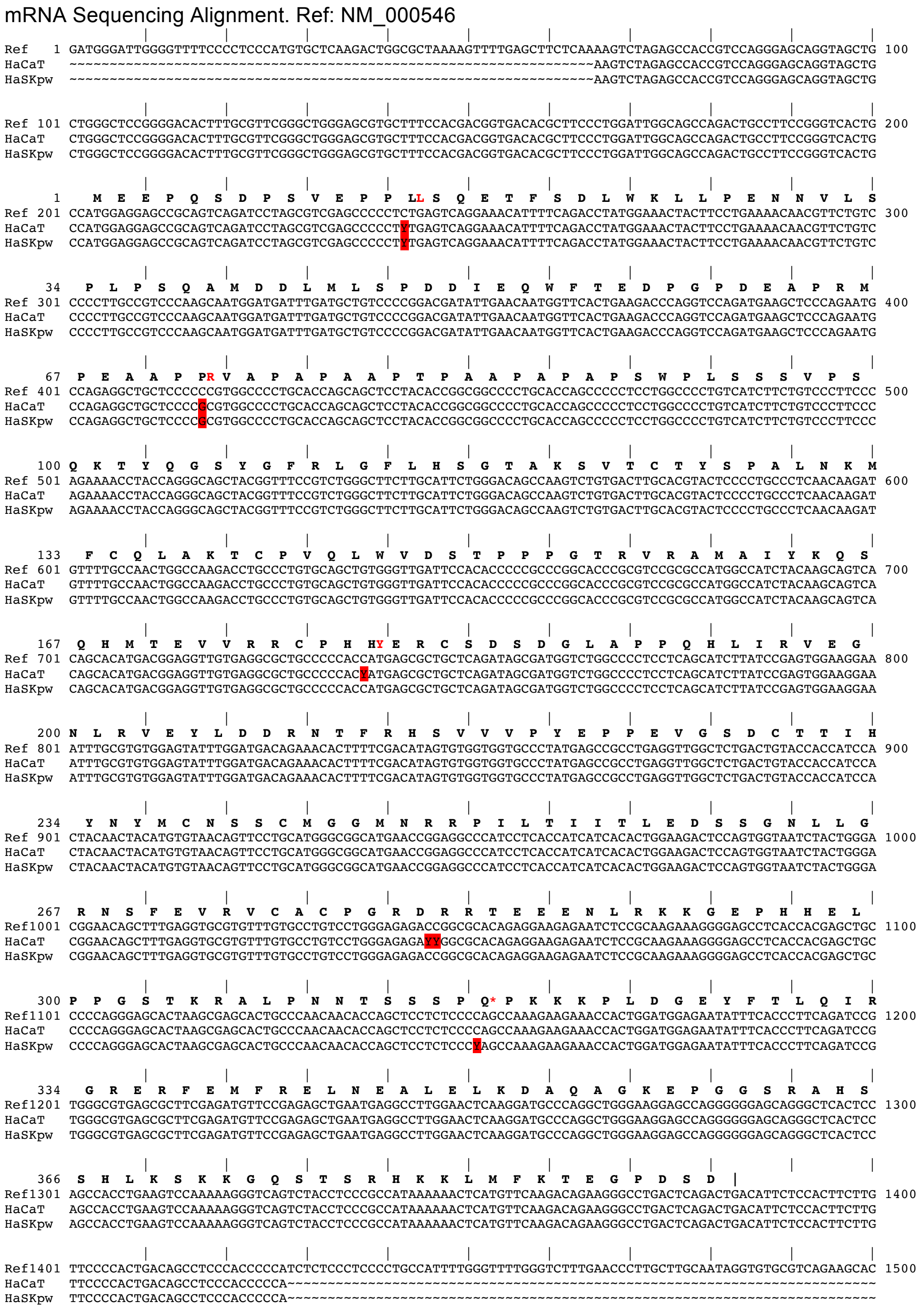

Figure S1: mRNA Sequence alignment of the p53 gene from HaSKpw and HaCaT. The consensus sequences of the mRNA sequencing results of the p53 gene from HaCaT and HaSKpw cells are aligned against the NM_000546. Mismatches are above the alignment.

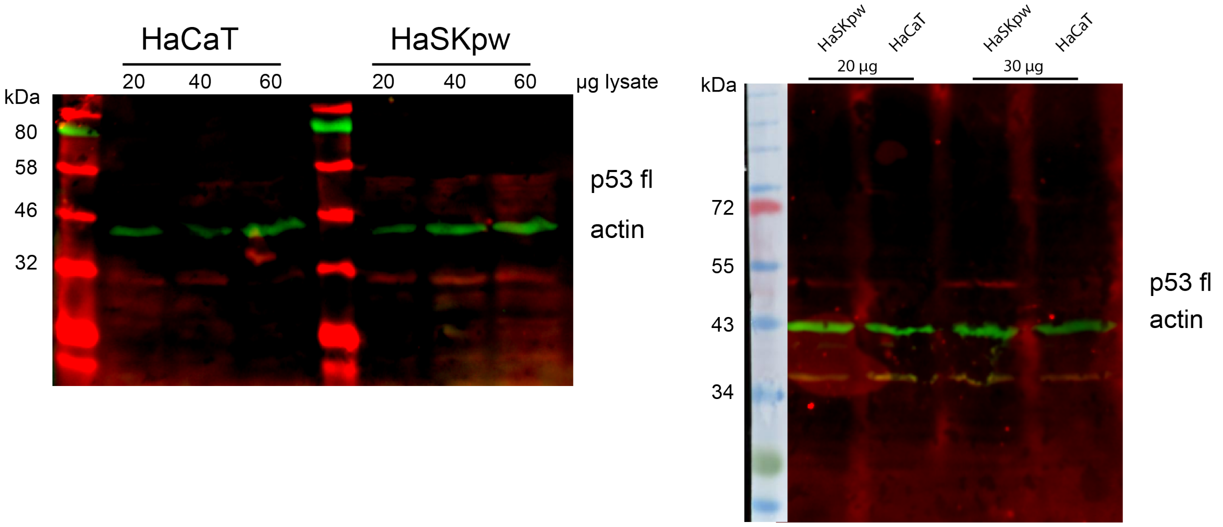

Figure S2: Western blot of HaSKpw and HaCaT cells for the detection of p53 protein. Indicated amounts of cell lysate are loaded and the p53 protein was detected with a rabbit-anti-p53 antibody (red). Actin was detected as loading control (green). Note that only the HaSKpw cells show full length of p53. Two biological replicates are shown.
